# Single-cell profiling identifies ACE^+^ granuloma macrophages as a nonpermissive niche for intracellular bacteria during persistent *Salmonella* infection

**DOI:** 10.1126/sciadv.add4333

**Published:** 2023-01-06

**Authors:** Trung H. M. Pham, Yuan Xue, Susan M. Brewer, Kenneth E. Bernstein, Stephen R. Quake, Denise M. Monack

**Affiliations:** ^1^ Department of Microbiology and Immunology, Stanford University, School of Medicine, Stanford, CA, USA.; ^2^ Department of Pediatrics, Stanford University School of Medicine, Stanford, CA, USA.; ^3^ Department of Bioengineering, Stanford University, Stanford, CA, USA.; ^4^ Department of Pathology and Laboratory Medicine, Cedars-Sinai Medical Center, Los Angeles, CA, USA.; ^5^ Chan Zuckerberg Biohub, San Francisco, CA, USA.

## Abstract

Macrophages mediate key antimicrobial responses against intracellular bacterial pathogens, such as *Salmonella enterica*. Yet, they can also act as a permissive niche for these pathogens to persist in infected tissues within granulomas, which are immunological structures composed of macrophages and other immune cells. We apply single-cell transcriptomics to investigate macrophage functional diversity during persistent *S. enterica* serovar Typhimurium (*S*Tm) infection in mice. We identify determinants of macrophage heterogeneity in infected spleens and describe populations of distinct phenotypes, functional programming, and spatial localization. Using an *S*Tm mutant with impaired ability to polarize macrophage phenotypes, we find that angiotensin-converting enzyme (ACE) defines a granuloma macrophage population that is nonpermissive for intracellular bacteria, and their abundance anticorrelates with tissue bacterial burden. Disruption of pathogen control by neutralizing TNF is linked to preferential depletion of ACE^+^ macrophages in infected tissues. Thus, ACE^+^ macrophages have limited capacity to serve as cellular niche for intracellular bacteria to establish persistent infection.

## Teaser

ACE+ macrophages have restricted capacity to act as a cellular niche that enables intracellular bacterial persistence.

## INTRODUCTION

Intracellular bacteria such as *Salmonella enterica* (*S. enterica*), *Brucella melitensis*, and *Mycobacterium tuberculosis* (*Mtb*) infect hundreds of millions of people and cause millions of deaths annually (*1*, *2*). These pathogens can establish persistent infection and survive in host tissues at low levels for months to years (*3*, *4*). Macrophages (MΦs) mediate key antibacterial immune responses, such as phagocytizing and killing bacteria, producing proinflammatory cytokines, and modulating adaptive immunity (*5*, *6*). Yet, MΦs also act as a cellular niche for intracellular bacteria to persist within the granulomas, which are tissue microstructures composed of MΦs and other cell types (*7*–*11*). Persistent intracellular bacterial infections pose great clinical challenges due to transmission from asymptomatic carriers, ineffective strategies for monitoring disease progression, and prolonged antimicrobial therapies that increase the risk for developing antimicrobial resistance (*12*). Modulating the differential functions of MΦs presents a viable therapeutic strategy to limit intracellular bacterial infections. However, our understanding of MΦ heterogeneity and its functional diversity in infected tissues remains largely incomplete.

Although invasive biopsy is not routinely performed, human histopathological studies suggest that MΦ-rich granulomas are a common feature of intracellular bacteria–infected tissues, such as spleens and livers (*13*–*15*). Animal infection models have been essential for understanding how diverse tissue MΦ functions and granuloma formation contribute to the persistence of bacilli. For example, persistent infection in systemic tissues such as the spleen has been investigated by infecting 129x1/SvJ mice with fully virulent *S. enterica* serovar Typhimurium (*S*Tm) (*3*, *9*, *10*). In chronically infected mice, *S*Tm bacilli persist for months at low abundance within splenic granulomas composed of heterogeneous MΦs. Neutrophils, lymphocytes, and other immune and nonimmune cells variably contribute to a spectrum of granuloma cellular composition, even among granulomas in the same infected tissue (*11*). Innate cellular antibacterial functions and T cell immune responses have been shown to be important for containing pathogens within intracellular bacterial granulomas and controlling infection (*16*). Recent studies suggest that the balance between proinflammatory, M1-like and anti-inflammatory, and M2-like MΦ activities within granulomas and intracellular bacteria–infected tissues influences bacterial persistence and eradication (*10*, *17*). How intracellular bacterial pathogens survive within granulomas despite MΦ recognition and antibacterial mechanisms, as well as robust innate and adaptive immune responses, remains a central question.

Approaches to defining diverse MΦ phenotypes and functions in infected tissues often depend on biased cellular features derived from a dichotomous paradigm of the classically activated (M1) and alternatively activated (M2) model (*18*–*21*). While overly simplified, M1-like MΦs are thought to be proinflammatory and antibacterial, whereas M2-like MΦs are thought to be more anti-inflammatory, pathogen permissive, and crucial for tissue repair. Increasing evidence suggests that the dichotomous M1 and M2 paradigm is insufficient to fully account for the heterogeneous phenotypes of MΦs in vivo (*22*). A multitude of factors, including ontogenetic programming, microenvironmental signals, and bacterial effector manipulation, shape tissue MΦ heterogeneity and functional diversity (*6*, *10*, *21*, *23*, *24*). MΦs localizing in different regions of granulomas within intracellular bacteria–infected tissues exhibit distinct phenotypes and may have differential functions such as antibacterial activities and T cell modulation (*9*, *25*). We and others previously showed that *S*Tm uses its *Salmonella* pathogenicity island 2 (SPI2) type 3 secretion system (T3SS) translocated effector E (SteE) to promote bacterial persistence within granulomas by skewing MΦ phenotype toward a permissive, M2-like state (*10*, *23*, *24*). During the persistent infection stage, many granuloma MΦs harboring intracellular *S*Tm also express high levels of the inducible nitric oxide synthase (iNOS), which has been used as a canonical marker of M1-like MΦs (*9*). Tissue MΦs have also been found to have different developmental origins that may influence their capacity to restrict intracellular bacteria. For example, in the lungs of *Mtb*-infected mice, monocyte-derived interstitial MΦs and alveolar MΦs, which originate from embryonic precursor cells, exhibit differential antibacterial capacities (*21*, *26*). Despite their ability to restrict intracellular bacilli, alveolar MΦs were shown to exhibit M2-like characteristics and thereby served as a more favorable replicative niche for *Mtb*. In a recent single-cell RNA sequencing (scRNA-seq) study of acute *S*Tm infection in mice for 48 hours after inoculation, when granulomas are yet to form and bacterial levels are uncontrolled, a non–classical monocyte (CM)–derived MΦ population was found to harbor more intracellular bacilli than other mononuclear phagocytes (MNPs) (*27*).

Defining MΦ heterogeneity in chronic intracellular bacterial infection in an unbiased manner is critical for understanding how MΦ functional diversity contributes to restricting microbes and limiting immunopathology yet enables the pathogens to persist at low levels within granulomas and infected tissues for long periods of time. Here, we performed scRNA-seq to determine the functional diversity that underlies the capacity of MΦs to permit or limit bacterial persistence in the spleens of mice that have been chronically infected with *S*Tm for 1 month. We identified determinants of MΦ heterogeneity and described populations of distinct phenotypes, functional programming, and spatial localization. Using the Δ*steE S*Tm mutant, which has a defect in counteracting host signals to skew MΦ phenotypes and maintain tissue persistence, we delineated MΦ phenotypes that contribute to controlling the infection. We found that angiotensin-converting enzyme (ACE) expression defines a splenic MΦ population that localizes to granulomas, and the abundance of the ACE^+^ MΦ niche anticorrelates with tissue bacterial burdens. ACE^+^ granuloma MΦs rarely harbor persistent *S*Tm, and granuloma MΦs with intracellular *S*Tm are much less likely to express ACE, indicating that ACE^+^ MΦs are a nonpermissive phenotype. *S*Tm entry into ACE^+^ MΦs is highly efficient and similar to other granuloma MΦ populations, suggesting that entry is not a limiting factor for *S*Tm to exploit ACE^+^ MΦs as a cellular niche for persistence. Disruption of pathogen control by neutralizing tumor necrosis factor (TNF) is linked to preferential and marked depletion of ACE^+^ MΦs in infected spleens compared to iNOS^+^ MΦs, which are a cellular niche for *S*Tm within granulomas. Collectively, our findings demonstrate that ACE^+^ MΦs are a functionally distinct phenotype that have limited capacity to serve as cellular niche for intracellular bacteria to establish persistent infection.

## RESULTS

### The spectrum of splenic macrophages, monocytes, and their precursors during persistent *S*Tm infection

scRNA-seq is a powerful approach for identifying cellular phenotypes and their functional roles in an unbiased fashion (*28*, *29*). We used scRNA-seq to comprehensively define MΦ heterogeneity and functional diversity in the spleens of 129x1/SvJ mice that were infected with fully virulent *S*Tm for 1 month, a time point at which the bacterial levels have been controlled, and persistent infection has been established (*3*, *10*). The SPI2 T3SS effector SteE polarizes MΦs to a more permissive state and thereby promotes *S*Tm persistence (*10*, *23*, *24*). As a result, mice infected with Δ*steE S*Tm mutant have approximately 10-fold less bacteria in the spleens by 1 month after inoculation compared to wild-type (WT) *S*Tm-infected mice (*10*, *30*). We performed scRNA-seq on splenocytes from both WT *S*Tm- and *ΔsteE S*Tm-infected mice to gain insights into how MΦ states and phenotypes control bacterial persistence (fig. S1A).

We previously showed that splenic *S*Tm granulomas are densely populated by CD11b^+^CD11c^+^Ly6C^+^ MNPs that express high levels of the phagocytosis receptor CD64, a marker universally expressed in MΦs across mouse tissues (*10*, *31*). These granuloma MΦs also have high levels of major histocompatibility complex II (MHC II) and F4/80 expression consistent with activated MΦs (fig. S1B) (*10*). The CD11b^+^CD11c^+^Ly6C^+^ granuloma MΦs (hereafter referred to as granuloma MΦs) are rare in the uninfected spleens but expand by 100-fold to constitute approximately 1% of total cells in the infected spleens by 1 month after inoculation (*10*). Thus, to comprehensively define the phenotypes and functional features of these granuloma MΦs, their precursors, and other types of MΦs in *S*Tm-infected spleens, we devised a permissive fluorescence-activated cell sorting (FACS) enrichment strategy that simultaneously enriches for granuloma MΦs and captures other splenocytes for droplet-based scRNA-seq using 10X Genomics platform (fig. S1, A and B) (Materials and Methods). We performed two independent experiments, each with two WT *S*Tm and two Δ*steE S*Tm-infected mice. We captured and sequenced a total of 40,281 cells. We filtered cells with low quality by removing those that had <1000 unique molecular identifier (UMI) counts, <500 detected genes, and >5% reads of mitochondrial origin, resulting in 22,512 cells that passed quality controls (fig. S1, D to G). We detected, on average, 2714 genes per cell and observed no substantial differences between individual experiments (fig. S1, D to G). We combined the filtered samples for downstream analysis.

**Fig. 1.**
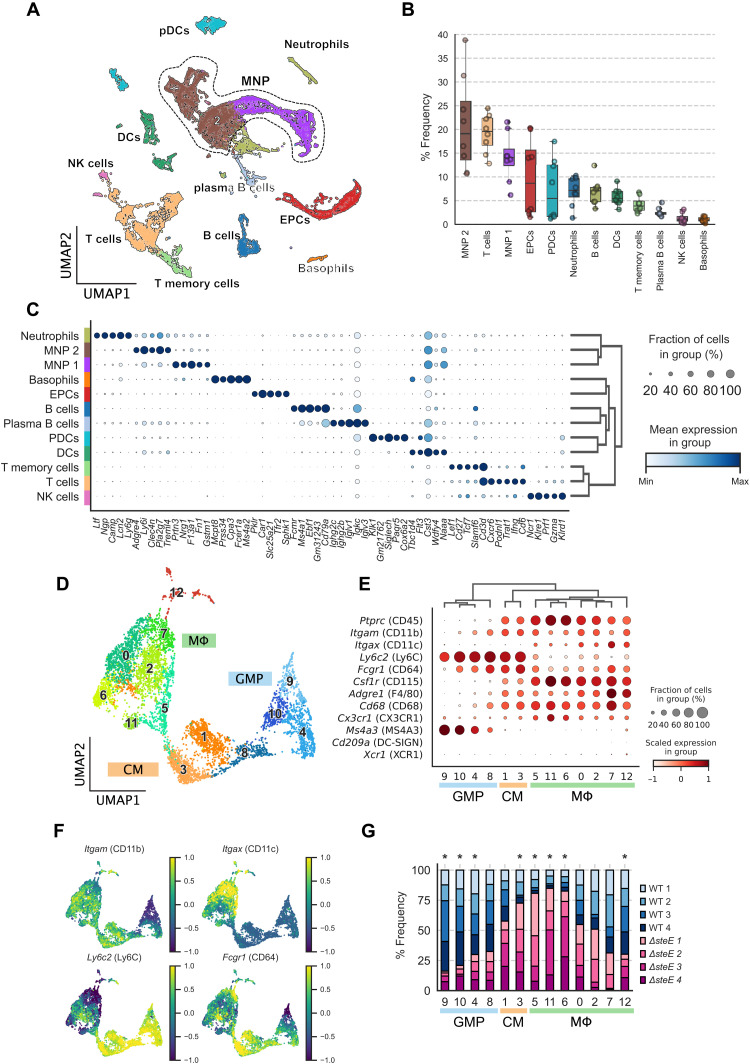
The spectrum of splenic macrophages, monocytes, and their precursors during persistent *S*Tm infection. Splenocytes from mice infected with either WT *S*Tm or Δ*steE S*Tm for 4 weeks were permissively FACS-sorted to simultaneously enrich for MΦs and capture all immune cell types for scRNA-seq. (**A**) Uniform Manifold Approximation and Projection (UMAP) of sequenced cells from both WT *S*Tm- and *ΔsteE S*Tm-infected spleens. DCs, dendritic cells; pDCs, plasmacytoid DCs; NK cells, natural killer cells; EPCs, erythroid precursor cells. (**B**) Percent frequencies for each immune cell type. (**C**) Top five DEGs in each immune cell population. Radius and color intensity of the dots reflect detection rate and mean expression for each gene, respectively. (**D**) UMAP projection of MNPs, excluding dendritic cells and neutrophils, colored by the assigned subpopulations: GMPs, CMs, and MΦs. (**E**) Dotplot showing expression levels of myeloid marker genes on GMP, CM, or MΦ clusters. (**F**) Expression levels of *Itgam* (CD11b), *Itgax* (CD11c), *Ly6c2* (Ly6C), and *Fcgr1* (CD64) previously shown to express on MΦs that densely populated *S*Tm granulomas (fig. S1B). (**G**) Differential representation test for GMP/CM/MΦ clusters in WT *S*Tm- and *ΔsteE S*Tm-infected animals. Asterisk above the bar indicates a greater than twofold difference in representation ratio and statistical significance in association with bacterial strain based on differential representation test (FDR < 0.05).

To resolve cellular heterogeneity in the dataset, we applied an unsupervised, soft feature-learning method, called self-assembling manifold (SAM) (*32*), to learn a cell-to-cell similarity matrix based on the intrinsic variation of gene expression between cells. On the basis of the resulting graph, we then inferred the cell types by clustering cells that share similar gene expression profiles (fig. S2A). We annotated the cell type and designated the immune cell types of our scRNA-seq dataset by referencing the scRNA-seq PanglaoDB database (*33*). We captured all the major splenic immune cell types, as expected by our permissive FACS enrichment strategy (Fig. 1, A to C). We found that each cell cluster contains a mixture of cells from different individual mice, suggesting that experimental batch effect is not a dominant source of variation in our dataset (fig. S2B). MΦs and monocytes, along with dendritic cells, are tissue sentinels that make up the MNP system (*34*). Tissue MΦs have dual developmental origins. In all mammalian tissues, a fraction of these cells originate from embryonic precursors and others share a direct lineage with monocytes (*22*). Notably, MNPs that are not dendritic cells constitute over 37% of the total cell population (Fig. 1, A and B), indicating that MΦs, monocytes, and their precursors are highly enriched in our dataset.

Next, we performed subclustering of the MNP groups (demarcated by dotted boundary in Fig. 1A) to investigate the spectrum and heterogeneity of MΦs, monocytes, and their precursors more in depth. Myeloid cells are highly heterogeneous and exhibit overlapping transcriptional landscapes (*31*). Among the MNP clusters, we identified multiple distinct populations of MΦs, CMs, and granulocyte-monocyte progenitors (GMPs), which gives rise to monocytes and monocyte-derived MΦs (Fig. 1D) (*35*). We validated the subcluster identities by performing hierarchical clustering on a panel of myeloid lineage marker and functional genes (Fig. 1E). Notably, the GMP clusters (GMPs 9, 10, 4, and 8) express high levels of the GMP marker *Ms4a3* (*36*). Compared to the GMP and CM clusters (CMs 1 and 3), the seven distinct MΦ clusters (MΦs 5, 11, 6, 0, 2, 7, and 12) express intermediate levels of *Ly6c2*. In addition, they coexpress varying levels of *Itgam* (CD11b), *Itgax* (CD11c), *Fcgr1* (CD64), *Adgre1* (F4/80), and *Csf1r* (CD115) (Fig. 1, E and F), indicating that they encompass the *S*Tm granuloma MΦs we previously identified (*10*). By contrast, the MΦ clusters express very low levels of *Cd209* and *Xcr1*, which are more highly expressed on dendritic cells.

To determine the impact of the SPI2 T3SS effector SteE on the functional heterogeneity of MΦs and MΦ precursors, we measured differential representation of GMP, CM, and MΦ phenotypes in WT *S*Tm-infected compared to Δ*steE S*Tm-infected samples (Fig. 1G) (Materials and Methods). We found that GMP clusters 4, 9, and 10 and MΦ 12 are significantly more abundant in WT *S*Tm-infected spleens [false discovery rate (FDR) < 0.05]. In contrast, CM cluster 3 and MΦ clusters 5, 6, and 11 are significantly more enriched in Δ*steE S*Tm compared to WT *S*Tm infection. These findings suggest that the SteE effector activity markedly altered the composition and functional diversities of MΦs, monocytes, and their precursors in infected tissues that may contribute to bacterial persistence and shape the infection.

### Delineating distinct macrophage functional programming and phenotypes in infected spleens

**Fig. 2.**
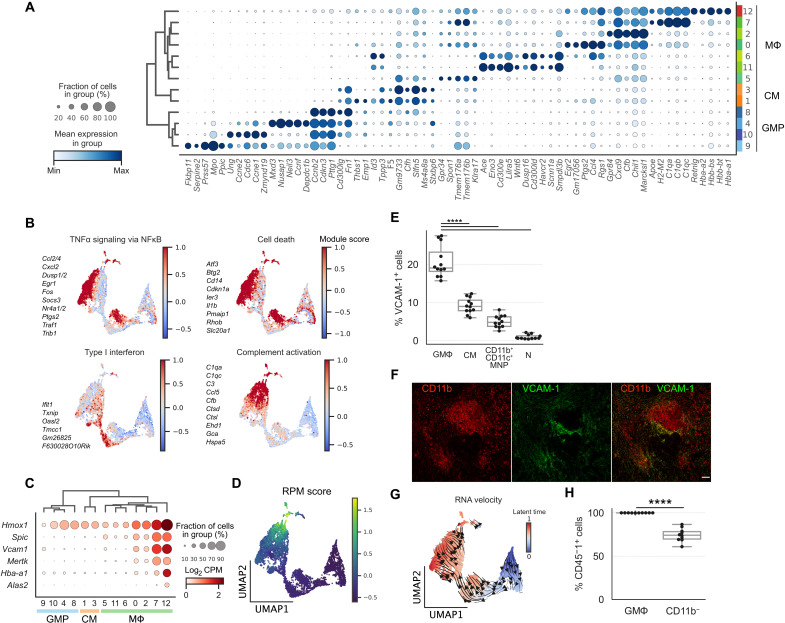
Delineating distinct macrophage functional programming and phenotypes in infected spleens. (**A**) Top five DEGs for GMP, CM, and MΦ clusters. Radius and color intensity of the dots reflect detection rate and mean expression for each gene, respectively. (**B**) Ensemble expression score of immune response gene sets using top DEGs (see Materials and Methods). Members of the gene sets are shown next to the plots. (**C**) Dotplot showing expression levels of RPM marker genes. (**D**) Ensemble expression score of RPM marker genes (*Hmox1*, *Spic*, *Vcam1*, *Mertk*, *Hba-a1*, and *Alas2*). (**E**) Frequencies of VCAM-1 expression in different myeloid populations in WT *S*Tm-infected spleens at 1 month after inoculation by flow cytometry. Granuloma MΦ (GMΦ), CM, CD11b^+^CD11c^+^ MNP, and neutrophil (N). (**F**) Confocal imaging of splenic granuloma MΦs stained for CD11b (red) and VCAM-1 (green). Scale bar, 50 μm. (**G**) UMAP projection of GMP, CM, and MΦ clusters with predicted RNA velocity vector field overlaid on the top hints at developmental transition from progenitor state to VCAM-1^+^ MΦs. (**H**) Percent frequencies of CD45-1^+^ cells among VCAM-1^+^ granuloma MΦs and CD11b^−^ cells in CD45.1^−^ recipient chimeric mice reconstituted with CD45.1^+^ bone marrow. (E and H) Dots: Individual mice. Significance calculated using a two-tailed Mann-Whitney test. ****P < 0.0001. (E) *n* = 12 mice, three independent experiments. (F) *n* = 6 mice, multiple sections per mouse, three independent experiments. (H) *n* = 10 mice, two independent experiments. TNFα, tumor necrosis factor–α; NFκB, nuclear factor κB.

Next, we used our single-cell transcriptomics analysis to determine the heterogeneity of MΦ populations with distinct functional states and phenotypes in the infected spleens. We performed differentially expressed gene (DEG) analysis and identified the most enriched gene sets for each of the GMP, CM, and MΦ clusters (Fig. 2A). This analysis demonstrates that these cell clusters are differentiated by distinct expression patterns of genes involved in MΦ functions and responses, including TNF signaling, cell death, type I interferon, and complement activation (Fig. 2B). These MΦ functional activities are crucial for host immune response against bacterial pathogens (*37*–*39*). Innate cellular responses are typically thought to be immediate and early host immune response against pathogens. Even at 1 month after inoculation when *S*Tm infection is fully established in the spleens, and the bacterial level has been controlled (*10*), we observed substantial heterogeneity in MΦ functional responses, suggesting that tissue MΦ immunity is highly heterogeneous during persistent infection in vivo and involves MΦs with varying antibacterial capacities.

In our analysis of the MΦ clusters, we found two clusters (MΦs 7 and 12) with uniquely high *Vcam1* expression (Fig. 2C). In the spleen, red pulp macrophages (RPMs), which are thought to originate from embryonic precursors, had been shown to express vascular cell adhesion molecule 1 (VCAM-1) (*40*, *41*). RPM development is dependent on the Spi-C transcription factor (SPIC), and these MΦs play a role in heme metabolism through phagocytosing spent red blood cells (*40*, *41*). To delineate the *Vcam1-*expressing MΦ clusters 7 and 12, we examined the expression of RPM marker genes, including *Spic*, *Hmox1*, *Vcam1*, *Mertk*, *Hba-a1*, and *Alas2* (Fig. 2C). We found that these genes are highly expressed in both MΦ clusters 7 and 12, except *Alas2*, which is detected only in MΦ cluster 12. We calculated an ensemble score (see Materials and Methods) based on the expression of RPM marker genes and identified MΦ cluster 12 as RPMs (Fig. 2D). As shown in [Fig. 1 (E and F)], *Vcam1^+^* MΦ cluster 7 also coexpresses CD11b (*Itgam*), CD11c (*Itgax*), Ly6C (*Ly6c2*), and CD64 (*Fcgr1*), suggesting that they may be MΦs that contribute to the formation of splenic granulomas during *S*Tm infection. To investigate this possibility, we performed flow cytometry analysis and found that VCAM-1 is expressed on approximately 20% of granuloma MΦs, while other cell types of myeloid lineages have lower expression (fig. S1B and Fig. 2E). By performing immunostaining on the *S*Tm-infected spleens, we found that VCAM-1^+^ granuloma MΦs are spatially localized to the periphery of granulomas (Fig. 2F).

The developmental origins of MΦs that organize into granulomas in intracellular bacteria–infected tissues are still not well defined (*42*). Prior studies showed that under steady state, bone marrow–derived monocyte precursors give rise to a population of VCAM-1^+^ splenic MΦs that regulate heme metabolism in the spleens (*43*). To test whether the VCAM-1^+^ granuloma MΦs in *S*Tm-infected spleens originate from a bone marrow origin, as opposed to embryonic precursors, we first performed RNA velocity analysis to gain insight into their development. Recent advances in scRNA-seq analysis have enabled the unbiased quantification of transcriptional trajectory based on kinetic modeling of mRNA splicing (*44*). To determine the transcriptional dynamics of *Vcam1*-expressing MΦ clusters, we applied RNA velocity to our scRNA-seq dataset. We determined an RNA velocity trajectory that is concordant with monocyte-MΦ transition and suggests that the *Vcam1*^+^ MΦ cluster 7 arises from bone marrow–derived GMPs (Fig. 2G). To validate this relationship, we generated bone marrow chimera by replacing the hematopoietic compartment in lethally irradiated CD45.2^+^ 129x1/SvJ recipient mice with bone marrow cells from CD45.1^+^ 129x1/SvJ donor mice. Fully reconstituted chimeric mice were then infected with WT *S*Tm and analyzed at 1 month after inoculation. We found that 100% of the VCAM-1^+^ granuloma MΦs were derived from CD45.1^+^ donors, demonstrating that they originated from an adult bone marrow source. By contrast, a lower fraction of the CD11b^−^ cells, which include radiation-resistant cells such as memory T cells, were derived from CD45.1^+^ donors (Fig. 2H). Collectively, these data demonstrate that our permissive FACS enrichment approach and single-cell transcriptomics identify a wide spectrum of tissue MΦs with distinct functional states, phenotypes, and ontogenetic development in the spleen during persistent *S*Tm infection.

### Identifying macrophage phenotypes associated with limiting infection

**Fig. 3.**
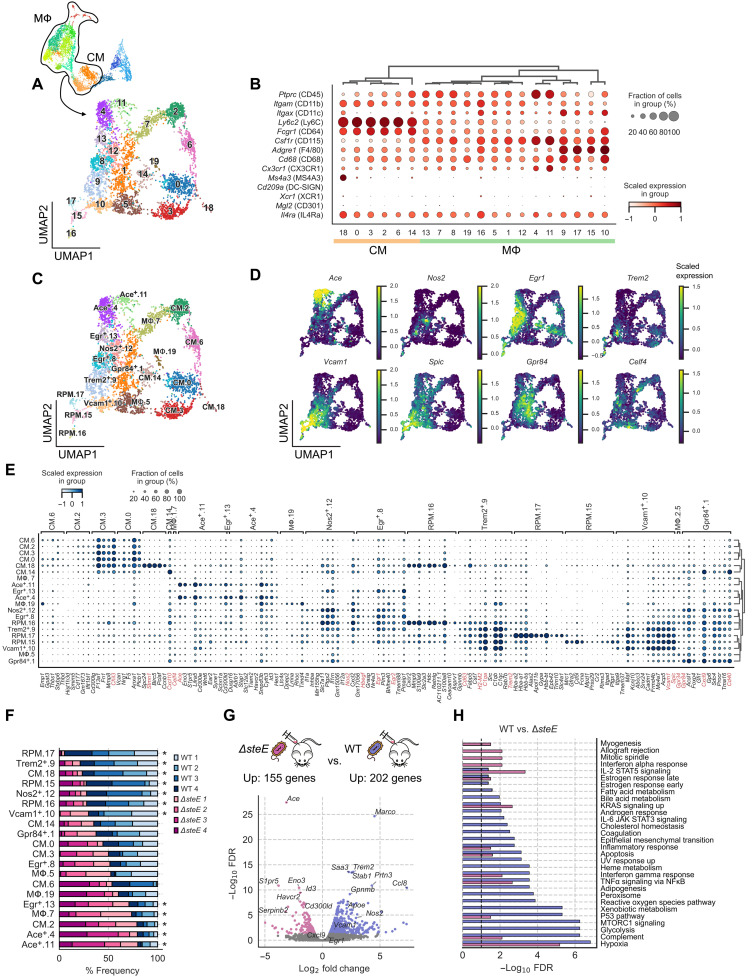
Identifying macrophage phenotypes associated with limiting infection. (**A**) UMAP projection of monocyte and MΦ subsets from WT *S*Tm- and Δ*steE S*Tm-infected mice. Cells are colored by Leiden cluster assignment. (**B**) Expression levels and frequencies of myeloid cell marker genes. (**C**) UMAP projection of monocyte and MΦ subsets from WT *S*Tm- and *ΔsteE S*Tm-infected mice. Cells are colored by cell state assignment. (**D**) Expression levels and frequencies of enriched marker genes of MΦ clusters: *Ace*, *Nos2*, *Egr1*, *Trem2*, *Vcam1*, *Spic*, *Gpr84*, and *Celf4*. (**E**) Expression levels and frequencies of the top five representative DEGs from each cell state (FDR < 0.05, expressed in >10% of cluster and log_2_ fold change > 0.5). A select panel of genes that define the functional state is highlighted in red. (**F**) Differential representation test for monocyte and MΦ states in WT *S*Tm- and *ΔsteE S*Tm-infected animals. Asterisk next to the bar indicates a greater than twofold difference in common odds ratio and statistical significance (<0.05 FDR) between the corresponding mice replicate (FDR < 0.05). (**G**) Volcano plot showing differentially gene expression analysis between combined MΦ populations with differential abundances [marked with asterisks in (F)] in WT *S*Tm- and *ΔsteE S*Tm-infected spleens. −Log_10_ FDR < 0.05 and log_2_ fold change > 0.5. Negative binomial test. (**H**) GSOA of the genes that are differentially up-regulated in the select MΦ populations [marked with asterisks in (F)] of WT *S*Tm- and *ΔsteE S*Tm-infected spleens. Genes with an FDR < 0.05, log_2_ fold changes > 0.5, and >10% expression in the corresponding group are selected as input for analysis. Bar width reflects the −log_10_ FDR. UV, ultraviolet; STAT3, signal transducers and activators of transcription 3; JAK, Janus kinase; MTORC1, mammalian target of rapamycin complex 1.

To further investigate the functional diversity and identify MΦ phenotypes that contribute to controlling persistent *S*Tm infection in the spleens, we subclustered the MNPs in Fig. 1D without the GMPs, which have high expression of cell cycle–related genes indicating proliferative activities (fig. S3, A and B). We identified 20 subclusters (Fig. 3A). Among these cells, clusters 18, 0, 3, 2, 6, and 14 exhibit transcriptional signatures consistent with CMs, whereas the remaining clusters were identified as MΦs by their patterns of myeloid lineage and functional marker expression (Fig. 3B). Using biased markers, we and others have previously found that MΦs, including granuloma MΦs, that are more permissive for *S*Tm persistence have higher levels of M2, or alternatively activated MΦ, markers such as interleukin-4Rα (IL-4Rα) and CD301 (*10*, *18*, *20*, *23*, *24*). However, it is unclear whether the expression of these markers defines a distinct MΦ transcriptional state or is enriched in a group of cells that are permissive to bacterial persistence from across different types of MΦs within the granulomas and infected tissues. In our scRNA-seq analysis, we found that *Il4ra* (IL-4Rα) and *Mgl2* (CD301) are not differentially expressed across most MΦ clusters, suggesting that these two genes alone are insufficient to define specific MΦ populations (Fig. 3B). These findings show that scRNA-seq transcriptomics can identify MΦ phenotypes and functional states that are not captured with existing approaches based on preselected markers.

We performed DEG analysis for each MΦ cluster and designated MΦ populations based on the top expressing genes (Fig. 3, C and D). Analysis of the topmost defining genes (FDR < 0.05, expressed in >10% of the cluster and log_2_ fold change > 1) for each annotated cluster reveals different MΦ phenotypes and functional states (Fig. 3E). Among these, we identified RPMs (clusters 15 to 17) and *Vcam1*^+^ granuloma MΦs (cluster 10), as characterized in Fig. 2. We identified additional functional heterogeneity in the MΦ subpopulations. Cluster 12 defines a MΦ population that expresses *Nos2*, which encodes iNOS, that had been previously shown to be a distinct granuloma MΦ phenotype in *Mtb*-infected lungs of nonhuman primates and *S*Tm-infected spleens of mice (*9*, *10*, *25*). *Trem2^+^* MΦs (cluster 9), which were recently identified as a distinct MΦ subset in human *Mycobacterium leprae* granulomas, are also present in our dataset (*45*). *Egr^+^* MΦs (clusters 8 + 13) express high levels of *Hbegf*, *Egr1*, and *Egr2*, which are early activating transcriptional factors in MΦs (*46*). Our analysis also identified *Vcan* as a highly enriched gene in the monocyte clusters (Fig. 3, C and E), which was shown to be a universal marker for monocytes across different human tissues based on scRNA-seq (*34*). MΦ clusters 1 and 7 express high levels of *Gpr84* and *Celf4*, respectively, although the expressions of these genes are relatively diffused across several MΦ subpopulations (Fig. 3, D and E). Intriguingly, we identified clusters 4 and 11 as MΦs that express high levels of *Ace*. ACE is a zinc-containing dipeptidyl carboxypeptidase that generates bioactive peptides regulating blood pressure, cardiovascular physiology, and inflammation (*47*). The role of ACE^+^ MΦs during persistent intracellular bacterial infections has not been defined.

The interactions between host signals and bacterial factors shape the balance between antibacterial and bacteria-permissive states in granuloma MΦs during infection (*10*, *17*, *23*). This balance affects bacterial control and infection outcome. The Δ*steE S*Tm mutant has a defect in polarizing MΦs toward a permissive state, leading to reduced bacterial tissue persistence and more rapid control of the infection (*10*, *23*, *24*). To identify MΦ phenotypes that may contribute to limiting bacterial persistence and infection, we compared the abundances of our scRNA-seq MΦ phenotypes in WT STm- and Δ*steE S*Tm-infected spleens. We performed a differential representation test to determine relative enrichment of monocytes and MΦs. We found that most of the frequency variation is in the MΦ subpopulations (Fig. 3F). Our analysis showed that Δ*steE S*Tm infection led to lower frequencies of RPM, *Nos2^+^*, *Vcam1^+^*, and *Trem2^+^* MΦs compared to WT *S*Tm infection. In contrast, the *Ace^+^* (clusters 4 and 11), *Celf4^+^* (cluster 7), and *Egr^+^* (cluster 13) subpopulations are more abundant in Δ*steE S*Tm-infected spleens, which have higher levels of bacterial clearance.

To gain insights into the impact of bacterial effector SteE activity on MΦ functional pathways, we focused on the MΦ populations that exhibited differential representation between WT *S*Tm- and Δ*steE S*Tm-infected animals (annotated with asterisks in Fig. 3F). Combining these populations into a WT *S*Tm and a Δ*steE S*Tm infection group, we performed DEG analysis to determine the impact of SteE activity on MΦ transcriptional programming (Fig. 3G). We identified 155 genes that are significantly up-regulated (FDR < 0.05 and log_2_ fold change > 0.5) in the MΦs from Δ*steE S*Tm-infected animals. The top DEGs are enriched in *Ace^+^* MΦs, including *Ace*, *Eno3*, *S1pr5*, and *Id3*, consistent with our representation analysis that *Ace^+^* MΦs were significantly enriched in *ΔsteE S*Tm-infected spleens. In contrast, 202 genes are significantly up-regulated in the MΦs from WT *S*Tm-infected animals, many of which are genes enriched in RPMs and *Nos2^+^* MΦs. We then quantified the enrichment of functional pathways by performing gene set overrepresentation analysis (GSOA) on the DEGs (see Materials and Methods). Despite robustly expressing genes involved in antibacterial responses such as TNF and interferon-γ signaling to a similar extent, splenic MΦs from the WT *S*Tm-infected mice are markedly more enriched in genes involved in complement activation, peroxisome, reactive oxygen species, glycolysis, heme metabolism, and adipogenesis, demonstrating remarkable SteE-driven effects on a wide range of MΦ immune and metabolic activities (Fig. 3H). Collectively, these results suggest that *Ace^+^* MΦs are one of the phenotypes that drive the functional transcriptional differences between MΦs in WT STm and Δ*steE S*Tm spleens, and their abundance is linked to infection containment.

### Splenic ACE^+^ macrophage population expands during infection and contributes to granuloma formation

**Fig. 4.**
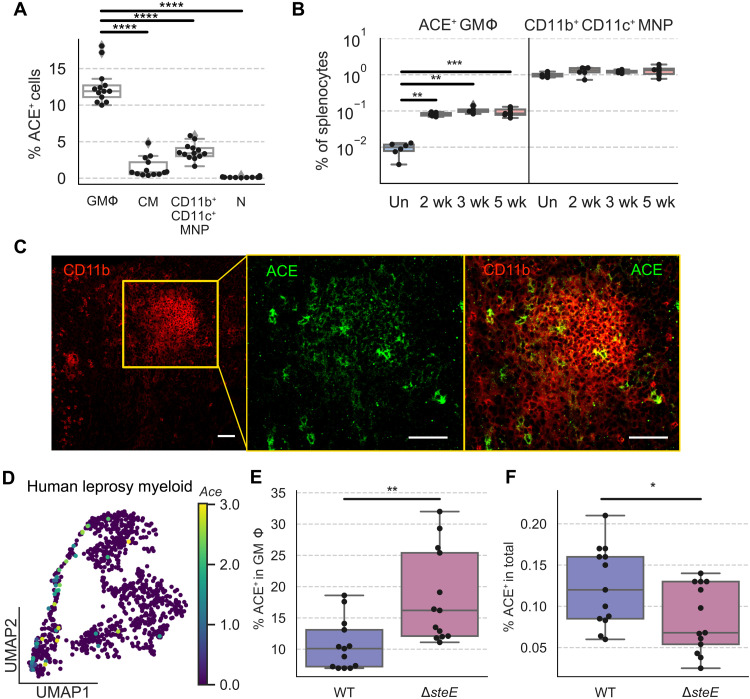
Splenic ACE^+^ macrophage population expands during infection and contributes to granuloma formation. (**A**) Mice were infected with WT *S*Tm and analyzed at 1 month after inoculation. Percent frequencies of ACE^+^ cells among GMΦs, CMs, CD11b^+^CD11c^+^ MNPs, and neutrophils (N) by flow cytometry. (**B**) Percent frequencies of ACE^+^ granuloma MΦs (left) and CD11b^+^CD11c^+^ MNPs (right) in total splenocytes at indicated time points after inoculation. Un, uninfected. (**C**) Immunofluorescence staining *S*Tm granuloma MΦs for CD11b (red) and ACE (green). Orange square on the left panel indicates the zoomed region shown in the two right panels. Scale bars, 50 μm. (**D**) UMAP projection of myeloid cells in human leprosy granulomas with scaled ACE expression overlaid on top. In these human myeloid cells, ACE expression is detected primarily in cluster 3 MΦ and cluster 2 *Trem2*^+^ MΦ (*45*). (**E** and **F**) Mice were infected with either WT *S*Tm or *ΔsteE S*Tm and analyzed at 1 month after inoculation by flow cytometry. (E) Percent frequencies of ACE^+^ cells among granuloma MΦs. (F) Percent frequencies of ACE^+^ granuloma MΦs among total splenocytes. (A, B, E, and F). Dots: Individual mice. Significance calculated using a two-tailed Mann-Whitney test. *P < 0.05, **P < 0.01, ***P < 0.001, and ****P < 0.0001. (A) *n* = 13 mice, three independent experiments. (B) *n* ≥ 6 mice per group, two independent experiments. (C) *n* = 5 mice, multiple sections per mouse, three independent experiments. (E and F) *n* = 13 mice per group, three independent experiments.

Our transcriptomics analyses suggest that *Ace^+^* MΦs are a phenotypically and functionally distinct population of MΦs in infected tissues during persistent *S*Tm infection. Intriguingly, ACE expression had been observed in human *Mtb* and sarcoidosis granulomas, suggesting that ACE^+^ MΦs may be commonly involved in granulomatous response across different persistent intracellular bacterial infections and pathophysiologic settings (*48*, *49*). However, whether ACE^+^ MΦs are a functionally distinct population of granuloma MΦs and what role they play during persistent intracellular bacterial infections remain unknown. To delineate *Ace*-expressing MΦs, we first identify them in infected tissues and determine their tissue dynamics during persistent *S*Tm infection in the spleens. We performed flow cytometry analysis and found that among cells of granuloma MΦ phenotype (fig. S1B), 10 to 20% express ACE, indicating that ACE^+^ MΦs are a subset of granuloma MΦs. The frequency of ACE^+^ cells are significantly lower among CM and CD11b^+^CD11c^+^ MNPs and are marginally above background staining among neutrophils (Fig. 4A and fig. S4, A to C). ACE^+^ MΦs are rare in uninfected spleens, but their frequency increased by more than 10-fold in the infected spleens by 2 weeks after inoculation (Fig. 4B), and their absolute number continues to rise through the third and fourth week of infection (fig. S4D). By contrast, the frequency of splenic CD11b^+^CD11c^+^ MNP remains relatively constant over the course of infection. To determine the spatial localization of ACE^+^ MΦs within *S*Tm splenic granulomas, we performed confocal microscopy and found that they are distributed throughout granulomas (Fig. 4C). These data demonstrate that ACE^+^ MΦs are a distinct tissue MΦ population that expands in response to *S*Tm infection and contributes to splenic *S*Tm granuloma formation.

Previous reports of ACE expression within human *Mtb* granulomas led us to investigate whether ACE is also expressed within distinct MΦ populations in other types of intracellular bacterial granulomas similar to what we observed in our murine granuloma model with persistent *S*Tm infection. Thus, we retrieved publicly available scRNA-seq data from a recently published study on *M. leprae* granulomas obtained from human skin biopsies (*45*). Our analysis of this scRNA-seq dataset found that *Ace* expression is detectable in at least two MΦ populations within the human *M. leprae* granulomas (Fig. 4D). Collectively, these findings suggest that ACE^+^ MΦs are involved in tissue granulomatous response across different intracellular bacterial pathogens, anatomical sites, and host species.

We next investigated whether the ACE^+^ granuloma MΦ phenotype is associated with *S*Tm infection outcome in the spleens by comparing their tissue levels in WT *S*Tm and Δ*steE S*Tm-infected mice, which have reduced bacterial persistence due to a bacterial defect in polarizing MΦ phenotypes (*10*). We found that by 1 month after inoculation, percent frequencies of ACE^+^ MΦs among granuloma MΦs were significantly higher in *ΔsteE S*Tm-infected spleens compared to WT *S*Tm-infected spleens (Fig. 4E). The percent frequencies of ACE^+^ granuloma MΦs among total splenocytes were slightly lower in the *ΔsteE S*Tm-infected spleens (Fig. 4F). By numbers, ACE^+^ granuloma MΦs decreased by 2.6-fold in Δ*steE* *S*Tm compared to WT *S*Tm-infected spleens, whereas total numbers of granuloma MΦs are reduced by 4.5-fold (fig. S4E). These findings suggest that the ACE^+^ phenotype is more abundant among granuloma MΦs in Δ*steE S*Tm-infected spleens, and this increased abundance may contribute to limiting *S*Tm persistence in the Δ*steE S*Tm-infected spleens.

### ACE^+^ granuloma macrophages are a nonpermissive cellular niche for *S*Tm

**Fig. 5.**
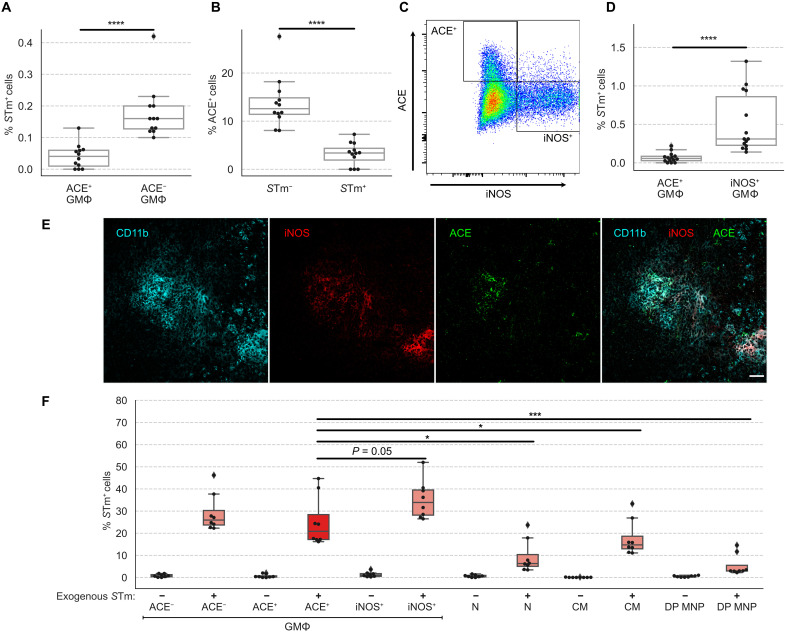
ACE^+^ granuloma macrophages are a nonpermissive cellular niche for *S*Tm. Mice were infected with WT *S*Tm and analyzed at 1 month after inoculation. (**A**) Percent frequencies of *S*Tm-infected cells among ACE^+^ and ACE^−^ granuloma MΦs by flow cytometry. (**B**) Percent frequencies of ACE^+^ cells among granuloma MΦs that are infected with *S*Tm. (**C**) ACE^+^ and iNOS^+^ granuloma MΦs form highly disparate populations. Splenocytes were analyzed by flow cytometry. Cells were first gated for granuloma MΦs as shown in fig. S1B. Then, ACE and iNOS expressions are shown. (**D**) Percent frequencies of *S*Tm-infected cells among ACE^+^ and iNOS^+^ granuloma MΦs by flow cytometry. (**E**) ACE^+^ and iNOS^+^ MΦs have overlapping distributions within granulomas. Immunofluorescence staining granuloma MΦs for CD11b (cyan), iNOS (red), and ACE (green) in splenic granuloma MΦs. Merged channels are shown on the far right panel. Scale bar, 50 μm. (**F**) Ex vivo measurements of *S*Tm entry into splenocytes from mice that had been infected for 1 month. Percent frequencies indicate entry of Tomato^+^ *S*Tm added exogenously ex vivo into different cell populations by FACS analysis. GMΦ, granuloma MΦ; N, neutrophil; DP MNP, CD11b^+^CD11c^+^ MNPs. (A, B, D, and F). Dots: Individual mice. Significance calculated using a two-tailed Mann-Whitney test. *P < 0.05, ***P < 0.001, and ****P < 0.0001. (A and B) *n* = 12 mice, three independent experiments. (D) *n* = 14 mice, three independent experiments. (E) *n* = 5 mice, multiple sections per mouse, three independent experiments. (F) Data pooled from three independent experiments, one to two mice per experiment with technical replicates.

We next investigated the potential relationship between ACE^+^ MΦs and *S*Tm persistence in infected tissues by determining the capacity for ACE^+^ MΦs to act as a cellular niche for intracellular *S*Tm. We measured the frequencies of *S*Tm-containing cells among these MΦs using flow cytometry. As expected with persistent *S*Tm infection in 129x1/SvJ mice, the pathogen is controlled at low chronic levels in the infected spleens by 1 month after inoculation, and approximately 0.2% of splenic granuloma MΦs contain intracellular bacteria at this stage of the infection (Fig. 5A) (*3*, *10*). However, we found that splenic ACE^+^ granuloma MΦs are markedly less likely to harbor intracellular *S*Tm compared to ACE^−^ cells (Fig. 5A). Furthermore, *S*Tm-containing granuloma MΦs were approximately fivefold more likely to have undetectable ACE expression compared to *S*Tm-negative granuloma MΦs (Fig. 5B). Collectively, these findings indicate that ACE^+^ granuloma MΦs are a nonpermissive niche for *S*Tm.

Although iNOS expression has commonly been associated with proinflammatory, antibacterial MΦs, we and others have observed that *S*Tm can persist within iNOS^+^ splenic granuloma MΦs even at 1 to 2 months after inoculation (fig. S5, A to C) (*9*, *10*). To see whether ACE^+^ and iNOS^+^ granuloma MΦs are phenotypically and functionally distinct populations, we analyzed splenocytes from infected mice for expression of these markers using flow cytometry. We found that ACE^+^ and iNOS^+^ cells form two largely nonoverlapping granuloma MΦ subsets (Fig. 5C). The frequency of iNOS^+^ granuloma MΦs infected with *S*Tm was eightfold higher than ACE^+^ granuloma MΦs (Fig. 5D). We then examined the spatial localization of ACE^+^ and iNOS^+^ MΦs to determine whether ACE^+^ MΦs were excluded from iNOS^+^ centers of granulomas, which was previously thought to be an underlying factor that CXCL-9/CXCL-10^+^ splenic MΦs are less likely to be infected with persisting *S*Tm (*9*). We found that ACE^+^ and iNOS^+^ granuloma MΦs have overlapping distributions within *S*Tm granulomas (Fig. 5E).

We next determined whether cellular entry contributed to the differences in intracellular *S*Tm levels seen among ACE^+^, ACE^−^, and iNOS^+^ granuloma MΦs. Because these cell populations are tissue MΦs that develop in infected spleens, we devised an ex vivo assay to assess *S*Tm entry into their intracellular compartments. We prepared single-cell suspension from the spleens of mice that have been infected with WT *S*Tm for 1 month, when the endogenous intracellular *S*Tm were ~1% or less among ACE^+^, ACE^−^, and iNOS^+^ granuloma MΦs (Fig. 5, A, B, and D). Suspended splenocytes were then subjected to ex vivo infection with Tomato-expressing *S*Tm for 30 min, and intracellular Tomato *S*Tm levels were quantified to measure entry. We observed that after 30 min, the intracellular Tomato^+^ *S*Tm levels of ACE^+^ and ACE^−^ and iNOS^+^ granuloma MΦs were significantly higher than neutrophils, CMs, and CD11b^+^CD11c^+^ MNP (Fig. 5F and fig. S5D). Tomato^+^ *S*Tm levels of ACE^+^ granuloma MΦs were similar to ACE^−^ granuloma MΦs and were marginally lower than iNOS^+^ MΦs (Fig. 5F). These data demonstrated that *S*Tm entry into different subsets of granulomas MΦs is highly efficient, and cellular entry is not likely a limiting factor for *S*Tm to exploit ACE^+^ MΦs as a cellular niche. Together, our data demonstrate that ACE^+^ granuloma MΦs are a distinct, nonpermissive MΦ niche for intracellular *S*Tm in infected tissues.

We then sought to determine whether ACE functionally controls the capacity of ACE^+^ MΦs to harbor intracellular *S*Tm. Transgenic mice (called ACE 10/10 mice) that overexpress *Ace* in myeloid cells due to ectopic placement of *Ace* under the *Csf1r* promoter have lower bacterial burdens during methicillin-resistant *Staphylococcus aureus* (*MRSA*) and *Listeria monocytogenes* (*L. monocytogenes*) infection at 3 to 5 days after inoculation (*50*, *51*). Peritoneal MΦs from ACE 10/10 mice exhibit an exaggerated proinflammatory response, with enhanced TNF, IL-6, and iNOS production, suggesting that ACE^+^ MΦs are more proinflammatory. In contrast, ACE-expressing human MΦs have been shown to have lower levels of proinflammatory cytokines such as TNF and IL-6 (*52*). Furthermore, intracellular bacteria that can cause persistent infection, such as *S. enterica* and *Bartonella henselae*, have mechanisms to modulate MΦ responses and skew MΦ phenotypes (*10*, *23*, *24*, *53*). Thus, the impact of ACE function on the capacity of MΦs to act as a cellular niche for intracellular bacterial persistence in infected tissues is unknown. To test whether ACE expression is sufficient to alter MΦ permissiveness to *S*Tm, we expressed ACE in RAW264.7 MΦs, which have undetectable ACE expression at baseline or during *S*Tm infection, using lentiviral transduction in vitro (fig. S5E). Although transduced MΦs robustly expressed ACE, the levels of intracellular WT *S*Tm were not affected (fig. S5E). We then sought to determine the impact of ACE overexpression in myeloid cells on *S*Tm infection in vivo. We crossed ACE 10/10 mice, which are of C57BL/6 background that is highly susceptible to WT *S*Tm, with 129x1/SvJ mice, which are able to control *S*Tm infection (*3*). We used the mixed background ACE 10 F1 offspring to perform persistent infection (fig. S5F). As expected, granuloma MΦs in the *S*Tm-infected spleens of ACE 10 F1 mice, which are heterozygous for *Ace 10* transgene, expressed significantly higher ACE levels compared to control F1 mice (fig. S5, F and G). However, both groups of mice have similar splenic bacterial levels at 2 weeks after inoculation (fig. S5H). Together, these findings suggest that ACE overexpression in MΦs alone is insufficient to affect *S*Tm persistence during in vitro and in vivo infection.

In addition, we tested to see whether inhibition of ACE enzymatic activity affects *S*Tm tissue persistence by infecting 129x1/SvJ mice with WT *S*Tm for 1 month and then treating them either with saline control or the ACE inhibitor captopril, 150 mg/kg per day intraperitoneally, for 7 days. This dose of captopril was previously shown to abolish approximately 95% of splenic ACE enzymatic activity in mice infected with *Histoplasma capsulatum* and is 30- to 50-fold higher than the maximum daily captopril dose used in treating cardiovascular disease in humans (*54*). We observed no significant difference in splenic bacterial levels from captopril treatment during persistent *S*Tm infection in vivo (fig. S5I). Collectively, our combined ACE overexpression and ACE enzymatic inhibition studies suggest that ACE is a defining marker of a nonpermissive MΦ niche for *S*Tm, and their capacity to act as a cellular niche for *S*Tm persistence is controlled by additional pathways beyond ACE.

### Disruption of pathogen control by TNF neutralization preferentially depletes ACE^+^ macrophages

**Fig. 6.**
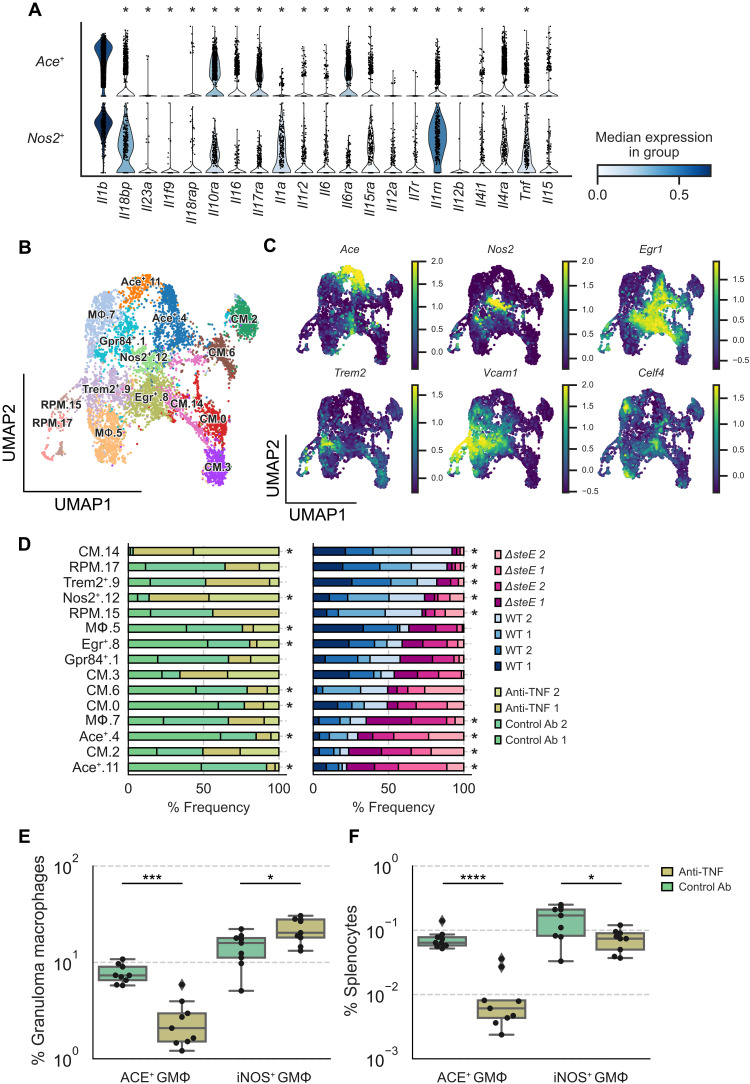
Disruption of pathogen control by TNF neutralization preferentially depletes ACE^+^ macrophages. (**A**) Violin plot highlighting cytokines that are differentially expressed between *Ace^+^* and *Nos2^+^* MΦs in WT *S*Tm- or *ΔsteE S*Tm-infected mice (see Fig. 3A) (FDR < 0.05). (**B**) Combined UMAP projection of scRNA-seq monocyte and MΦ subsets from mice that were treated with isotype control or anti-TNF antibody. Cells are colored by cell state assignment. (**C**) Expression levels of enriched marker genes of MΦ clusters: *Ace*, *Nos2*, *Egr1*, *Trem2*, *Vcam1*, and *Celf4*. (**D**) Differential representation test for monocyte and MΦ clusters in WT *S*Tm-infected mice that have been treated with isotype control or anti-TNF antibody (left) or mice infected with WT *S*Tm or *ΔsteE S*Tm (right). Asterisk next to the bar indicates a greater than twofold difference in representation ratio and statistical significance between treatments based on a differential representation test (FDR < 0.05; see Materials and Methods). Mice were chronically infected with WT *S*Tm for 1 month, treated with isotype control or anti-TNF antibodies on day 1, and analyzed by flow cytometry or prepared for scRNA-seq on day 4. (**E**) Percent frequencies of ACE^+^ and iNOS^+^ cells among splenic granuloma MΦs from animals treated with isotype control and anti-TNF antibody. (**F**) Percent frequencies of ACE^+^ and iNOS^+^ granuloma MΦs in total splenocytes from isotype control and anti-TNF–treated animals. (E and F) Dots: Individual mice. Significance calculated using a two-tailed Mann-Whitney test. *P < 0.05, ***P < 0.001, and ****P < 0.0001. (E and F) *n* = 9 mice per group, two independent experiments. Ab, antibody.

Our data indicate that ACE expression defines a distinct granuloma MΦ niche that is nonpermissive for *S*Tm, and this MΦ population has notably disparate capacity to harbor intracellular *S*Tm compared to iNOS^+^ granuloma MΦs during persistent infection (Fig. 5, A to D). Concordant with their phenotypic difference, unbiased pathway analyses revealed differential enrichment of genes involved in multiple pathogen response programming such as TNF signaling, type I interferon response, fatty acid metabolism, and hypoxia between *Ace^+^* MΦs and *Nos2^+^* MΦs (fig. S3C). Thus, to gain further insights into the cellular features and functional pathways underlying the phenotype of ACE^+^ MΦs and their regulations, we leveraged our transcriptomics data and compared the functional features of ACE^+^ MΦs with those of iNOS^+^ MΦs. We focused on cytokine and cytokine receptor expression, as cytokine signaling pathways—including IL-4/IL-13, IL-10, IL-1, TNF, IL-18, and IL-6—are key determinants of MΦ immune and metabolic states, antibacterial functions, and capacity to permit intracellular bacterial persistence (*10*, *23*, *24*, *30*, *53*). We found that *Ace^+^* and *Nos2^+^* MΦs exhibit remarkably different cytokine and cytokine receptor gene expression patterns. While having similarly robust *Il1b* expression, the *Ace^+^* MΦs express significantly lower levels of *Tnf*, *Il6*, and *Il15ra*, which mediate proinflammatory, antibacterial immune responses (Fig. 6A). However, compared to *Nos2^+^* MΦs, *Ace^+^* MΦs also express markedly less *Il18bp* and *Il1rn*, both of which encode natural antagonists that limit the IL-18 and IL-1 proinflammatory effects, respectively (*55*, *56*). *Ace^+^* MΦs express similar levels of *Il4r*α, a commonly used marker for anti-inflammatory, alternatively activated MΦs, but significantly higher levels of *Il10r*α compared to *Nos2^+^* MΦs (Fig. 6A). In contrast, *Ace^+^* MΦs also express higher levels of *Il6r*α and *Il17r*α, which transmit antimicrobial, proinflammatory signals. Collectively, our transcriptomics analyses demonstrate that *Ace^+^* and *Nos2^+^* MΦs have remarkably distinct transcriptional programming, with many contrasting cellular features that underlie their differential phenotypes and their capacity to harbor *S*Tm, and suggest that they may be divergently regulated to control bacterial persistence in infected tissues.

Our data demonstrate that ACE^+^ granuloma MΦs are a nonpermissive cellular niche for *S*Tm to persist within (Fig. 5, A and B), and the abundance of this MΦ phenotype is significantly higher in Δ*steE S*Tm-infected spleens (Fig. 4E), which have reduced bacterial tissue persistence (*10*, *30*). This led us to wonder whether increased splenic *S*Tm persistence and loss of pathogen restriction may be associated with depletion of ACE^+^ granuloma MΦs. To probe the relationship between splenic ACE^+^ MΦ abundance and *S*Tm tissue bacterial levels, we disrupted pathogen control in infected mice by neutralizing TNF, which we had shown previously leads to increased *S*Tm splenic levels by more than 10-fold (*10*). The TNF neutralization effect was mechanistically linked to skewing MΦs toward a more bacteria-permissive state and the loss of proinflammatory MΦ phenotypes (*10*, *57*). Thus, we treated mice that had been infected with *S*Tm for 1 month with either control or anti-TNF–neutralizing antibody. Because we observed notable differential enrichment of TNF signaling genes between *Ace^+^* and *Nos2^+^* MΦs (fig. S3C), we examined both of these MΦ populations to see whether TNF differentially affects *Ace^+^* and *Nos2^+^* MΦ abundance to control *S*Tm tissue persistence. Splenocytes were enriched for MΦs and monocytes using permissive FACS enrichment strategy as described (see Materials and Methods). Samples from two control antibody-treated and two anti-TNF antibody-treated animals were subjected to scRNA-seq. We combined sequenced single-cell transcriptomes from the antibody-treated animals with the transcriptomes obtained from the WT *S*Tm and Δ*steE S*Tm infection experiments (Fig. 3A) for a direct comparison of the same cell types. We observed that all MΦ and monocyte clusters, including the *Ace^+^* and *Nos2^+^* MΦ populations, were composed of cells from all animals across experiments (Fig. 6, B and C, and fig. S6A), suggesting a lack of experiment-specific batch effect. As expected, differential representation test showed that *Ace^+^* MΦs were more abundant in Δ*steE S*Tm-infected spleens than in WT *S*Tm-infected spleens (Fig. 6D). *Ace^+^* MΦs were more markedly depleted in the spleens of animals treated with TNF-neutralizing antibody compared to animals treated with control antibody (Fig. 6D). In contrast, *Nos2^+^* MΦs were significantly more abundant. To experimentally test whether TNF neutralization disparately affects the *Ace^+^* and *Nos2^+^* MΦ niches in infected spleens, we performed flow cytometry analysis of *S*Tm-infected mice that have been treated with either control or TNF-neutralizing antibody. We found that among splenic granuloma MΦs, the percent frequencies of ACE^+^ cells were reduced by fourfold in anti-TNF–treated mice compared to control antibody-treated mice. In contrast, the percent frequencies of iNOS^+^ cells increased slightly by 1.6-fold (Fig. 6E). Strikingly, the percent frequencies of ACE^+^ granuloma MΦs among total splenocytes were reduced by almost 10-fold in anti-TNF–treated mice compared to control mice, whereas the percent frequencies of iNOS^+^ granuloma MΦs were reduced by only twofold (Fig. 6F). In addition, we found that the antibody neutralization of TNF decreased the numbers of both ACE^+^ and iNOS^+^ granuloma MΦs in infected animals compared to isotype antibody treatment (fig. S6B). However, the ACE^+^ MΦ number decreased by approximately 13-fold, whereas the iNOS^+^ MΦ number decreased by less than twofold. Together, our data suggest that loss of pathogen control in infected spleens by TNF neutralization is linked to preferential and marked depletion of the nonpermissive, ACE^+^ granuloma MΦs.

## DISCUSSION

The tissue persistence of intracellular bacteria such as *S. enterica*, *Brucella* species, and mycobacteria hinders treatment effectiveness and facilitates spread of infections (*7*, *8*, *12*). Despite robust innate and adaptive immune responses, in many individuals, these pathogens can remain in infected tissues at low chronic levels for long periods of time, and the hosts may not exhibit overt clinical symptoms. Central to this type of bacterial tissue persistence is the functional diversity and heterogeneity of MΦs and MΦ granulomas (*9*, *10*, *16*, *26*, *58*). In infected tissues, MΦs mediate critical antibacterial immune responses that contribute to pathogen eradication, resolution of inflammation, and tissue repair. Yet, these crucial immune cells can also act as a cellular niche and form granulomas, which are immunological structures that function as a host mechanism to contain infection but within which intracellular bacteria are able to survive. Understanding of MΦ functional diversity requires delineation not only of MΦs within granulomas but also of precursor cells that give rise to these and other MΦs in the infected tissue environment (*26*, *27*, *45*, *58*). Here, we apply single-cell transcriptomics to investigate MΦ heterogeneity in *S*Tm-infected spleens to gain insight into how their heterogeneity and functional diversity contribute to controlling bacterial persistence and infection. Our permissive FACS enrichment strategy not only enables substantial enrichment of rare MΦ populations in infected tissues, such as the CD11b^+^CD11c^+^Ly6C^+^ granuloma MΦs, but also captures a full spectrum of splenocytes to facilitate comprehensive mapping of the tissue MΦ phenotypes during persistent infection (Figs. 1 and 2). In addition, we detected more than 2700 genes per cell, on average, which provides substantial sequencing depth for delineating differences among MΦ populations. Our single-cell transcriptomics dataset captures RPMs, a known type of MΦs in the spleen. We also identify *Trem2^+^* MΦs, which were recently identified as a MΦ subset in human *M. leprae* granulomas (*45*), as well as *Nos2^+^* MΦs, a distinct MΦ phenotype in *Mtb* and *S. enterica* granulomas (*9*, *10*, *25*). Our single-cell transcriptomics enable delineation of MΦ phenotypes in granulomas and infected tissues during persistent intracellular bacterial infection that have not been defined, such as the bone marrow–derived VCAM-1^+^ granuloma MΦ population (Fig. 2) and ACE^+^ granuloma MΦ population (Fig. 3).

While ACE expression in *Mtb* granulomas had been well described, whether ACE^+^ MΦs are a distinct subset of MΦs in granulomas and what their functions are during persistent intracellular bacterial infection remain unknown. Our single-cell transcriptomics and functional characterization demonstrate that ACE expression specifies a MΦ population that has distinctive cellular features, functional properties, and TNF regulation compared to other types of MΦs in *S*Tm granulomas and infected spleens (Figs. 4 to 6). The ACE^+^ granuloma MΦs described here are characterized by their expression of lineage markers CD11b, CD11c, and Ly6C, which is expressed at lower level compared to CMs (fig. S1, B and C), suggesting that they may be monocyte-derived MΦs (*59*). It is now well recognized that in almost all tissues, some MΦs originate from blood-borne monocytic precursors recruited to the tissues during steady state or in the setting of inflammation, whereas other MΦs arise from embryonic origin during development (*22*). Intriguingly, *Mtb* granulomas in various tissues in human and nonhuman primates are also populated with CD11b^+^CD11c^+^ MΦs (*25*, *60*), raising the possibility that these MΦs might be analogous to the CD11b^+^CD11c^+^Ly6C^+^ granuloma MΦs we have described. In addition, the ACE expression detected in human tuberculosis granulomas may reflect a functional equivalent ACE^+^ MΦ subset to the ACE^+^ granuloma MΦs that we report in this study. Our analysis of scRNA-seq dataset on *M. leprae* granulomas isolated from human skin biopsy demonstrates that *Ace^+^* MΦs are distinct granuloma MΦ phenotypes in human mycobacterial granulomas (Fig. 4) (*45*). Furthermore, ACE expression has been observed in granulomas in the autoimmune disease sarcoidosis (*49*). Collectively, these reports and our present study suggest that ACE^+^ MΦs might be involved in tissue granulomatous response across different types of tissues and diseases.

We found in this study that ACE^+^ MΦs in *S*Tm-infected spleens are less likely to harbor intracellular *S*Tm, and conversely, *S*Tm-containing MΦs rarely have detectable ACE expression, indicating that ACE^+^ MΦs are nonpermissive cellular niche for *S*Tm during persistent infection (Fig. 5, A to D). By not harboring intracellular bacteria such as other MΦs in granulomas and infected tissues, ACE^+^ MΦs may help limit the tissue persistence of *S*Tm. Our findings that the abundance of ACE^+^ phenotype among granuloma MΦs is higher in Δ*steE S*Tm-infected spleens, which have reduced bacterial persistence, but lower in spleens of TNF-neutralized animals, which have increased bacterial tissue levels and uncontrolled infection, suggest a link between ACE^+^ MΦ abundance and controlling bacterial persistence and infection. During persistent intracellular bacterial infection, diverse MΦs in infected tissues exhibit highly differential responses. Some MΦs kill intracellular bacteria, undergo cell death to eliminate intracellular bacteria, and promote antimicrobial activities of other immune cells. Others play a role in resolving inflammation that may indirectly enable bacterial persistence, act as a replicative niche with high levels of intracellular bacteria, or serve as permissive niche for pathogen to persist at low intracellular levels. Thus, the relative levels of these different MΦ functional states and phenotypes within granulomas and infected tissues influence bacterial control and shape infection outcome (*10*, *16*, *17*). Our finding that *S*Tm entry into ACE^+^ MΦs is highly efficient and similar to ACE^−^ and iNOS^+^ MΦs suggest that cellular entry is not a limiting factor for *S*Tm to exploit ACE^+^ MΦs as a cellular niche. Dissecting nonmutually exclusive possibilities that ACE^+^ MΦs have unfavorable intracellular metabolic environment for *S*Tm to survive, are more prone to undergo cell death, and/or quickly differentiate into a more antibacterial phenotype upon being infected will be interesting subjects for further studies in the future.

Our data indicate that ACE expression is a marker of a distinct MΦ functional state or phenotype that has restricted capacity to permit intracellular *S*Tm persistence in infected tissues. Prior studies using *Ace* transgenic mice showed that overexpression of ACE in *Csf1r*-expressing myeloid cells resulted in enhanced clearance of *MRSA* and *L. monocytogenes* from infected tissues at 3 to 5 days after inoculation (*51*). In our persistent *S*Tm infection model, gain-of-function and loss-of-function manipulations of the ACE pathway bear no significant impact of bacterial persistence during in vitro or in in vivo infection (fig. S5). We suspect that unlike *MRSA* and *L. monocytogenes*, vacuolar intracellular bacteria that cause persistent infection such as *S*Tm have a number of bacterial effector mechanisms to modulate MΦ responses and skew MΦ phenotypes (*9*, *10*, *23*, *24*). Future studies involving selective deletion of *Ace* in tissue MΦs will further clarify on a potential impact of the *Ace* pathway on the nonpermissiveness of ACE^+^ MΦs during persistent *S*Tm infection.

While ACE expression is a defining feature of a nonpermissive MΦ niche, our data suggest that additional ACE-independent pathways may influence the capacity of ACE^+^ MΦs to harbor *S*Tm. By using ACE expression as a marker, we have been able to track and interrogate the differential cellular features and functional properties of ACE^+^ granuloma MΦs to gain insights into their functions and regulation. We found that ACE^+^ and iNOS^+^ granuloma MΦs have vastly different capacities to harbor *S*Tm (Fig. 5). The nonpermissive, ACE^+^ phenotype is likely a composite functional outcome of many cellular pathways that affect MΦ antibacterial activities and intracellular bacterial persistence. Examination of the cytokine signaling gene expressions showed that *Ace^+^* MΦs in *S*Tm-infected tissues exhibit a pattern of cytokine and cytokine receptor expressions that does not neatly fit into either a proinflammatory or anti-inflammatory categorization (*51*, *52*). Compared to *Nos2^+^* MΦs, *Ace^+^* MΦs express less *Tnf*, *Il1a*, *Il6*, and *Il15r* but more *Il6r* and *Il17r*, all of which are associated with proinflammatory signaling (Fig. 6). On the other hand, they also express higher levels of *Il10r*, which mediates an anti-inflammatory signal. Intriguingly, a recent study demonstrated that human MΦs lacking *Il10r* unexpectedly had reduced ability to restrict intracellular *S*Tm (*61*). Their findings suggest that MΦ *Il10r* expression is associated with a more bactericidal state with different cellular metabolic activities, including altered prostaglandin levels, which are unfavorable for intracellular *S*Tm persistence (*61*). In addition, we observed that *Ace^+^* MΦs have markedly lower *Il18bp* and *Il1rn* expressions compared to *Nos2^+^* MΦs (Fig. 6). Deficiency of IL-18bp and IL-1Rn are thought to cause exaggerated proinflammatory responses in monocytes and MΦs and contribute to the development of inflammatory disorders such as MΦ activation syndrome and autoimmune arthritis (*55*, *56*). Corresponding with their differential cellular features, we found that TNF neutralization resulted in a preferential depletion of ACE^+^ MΦs (Fig. 6). TNF is a highly pleiotropic cytokine that has been linked to restraining the emergence of bacteria-permissive MΦ phenotypes and modulating MΦ cell death (*10*, *57*, *62*, *63*). We speculate that the differential signaling state of ACE^+^ MΦs may result in disparate impacts on the fate of these cells upon TNF neutralization compared to other splenic MΦs. Collectively, our findings of ACE^+^ MΦs reflect the multitude of factors that shape MΦ phenotypes and their functions during persistent intracellular bacterial infection. They also illustrate how single-cell transcriptomics provide fuller pictures of cellular functional features that underlie the overall MΦ phenotypes and functional diversity.

## MATERIALS AND METHODS

### Ethics statement

Experiments involving animals were performed in accordance with National Institutes of Health guidelines, the Animal Welfare Act, and U.S. Federal Law. All animal experiments were approved by the Stanford University Administrative Panel on Laboratory Animal Care and overseen by the Institutional Animal Care and Use Committee under protocol ID 12826. Animals were housed in a centralized research animal facility accredited by the Association of Assessment and Accreditation of Laboratory Animal Care International.

### Mouse strains and husbandry

Females and males 129X1/SvJ mice were obtained from the Jackson Laboratory (catalog no. 00691) or an in-house 129X1/SvJ colony. CD45.1^+^ 129x1/SvJ mice were generated from backcrossing CD45.1^+^ C57BL/6J mice (the Jackson Laboratory) to 129x1/SvJ mice (the Jackson Laboratory) successively for more than 10 generations. ACE 10/10 C57BL/6 mice were provided by K. Bernstein. Male and female mice (7 to 16 weeks old) were housed under specific pathogen–free conditions in filter top cages that were changed bimonthly by a veterinary or research personnel. Sterile water and food were provided ad libitum. Mice were given at least 1 week to acclimate to the Stanford Animal Biohazard Research Facility before experimentation.

### Bacterial strains and growth conditions

*S*Tm strain SL1344 was used in this study. SL1344 Δ*steE* and SL1344 *Tomato* were generated as described previously (*9*, *24*). For all mouse infections, *S.* Typhimurium strains were maintained aerobically on LB agar supplemented with streptomycin (200 μg/ml) and kanamycin (±40 μg/ml) and grown aerobically to stationary phase overnight at 37°C with broth aeration. Bacterial cultures were spun down and washed with sterile phosphate-buffered saline (PBS) before suspension in PBS for infection.

### Mouse infections and TNF neutralization

Mice were allocated to control and experimental groups randomly, sample sizes were chosen on the basis of previous experience to obtain reproducible results, and the investigators were not blinded. Mice were inoculated intraperitoneally with 1 to 2 × 10^3^ colony-forming units (CFU) *S.* Typhimurium SL1344 WT or Δ*steE* in 200 μl of PBS. For TNF neutralization, infected mice were injected intraperitoneally with either 500 μg of anti-TNF monoclonal antibody, clone MP6-XT22 (BioLegend) or isotype control antibody in sterile PBS in 400-μl total volume before scRNA-seq or analysis 3 days later. Mice were euthanized at the indicated time points after inoculation by CO_2_ asphyxiation followed by cervical dislocation as the secondary method of euthanasia. Organs were collected, weighted, and either homogenized in PBS for CFU enumeration, used to make single-cell suspension for flow cytometric analysis, or prepared for microscopy examinations.

### Bone marrow chimera

CD45.2^+^ 129x1/SvJ mice at 6 to 8 weeks of age were lethally irradiated (6 Gy twice, 6 hours apart). Single-cell suspension from bone marrows of CD45.1^+^ 129x1/SvJ donor mice was prepared and injected intravenously into irradiated CD45.2^+^ mice via tail vein. Each recipient mouse received 1 × 10^6^ to 2 × 10^6^ of donor bone marrow cells. Recipient mice were maintained for 2 weeks on autoclaved food and water containing neomycin sulfate (2 mg/ml; VWR 89149-866) and polymyxin B (1000 U/ml; MilliporeSigma P4932-5MU). Bone marrow engraftment was assessed ~8 weeks after transplantation. Mice were infected with *S.* Typhimurium SL1344 9 to 10 weeks after transplantation and analyzed 1 month after inoculation.

### Flow cytometry

Spleens from mice were minced with surgical blades no. 22 and incubated in digestion buffer [Hanks’ balanced salt solution (HBSS) + Ca^2+^ + Mg^2+^ + deoxyribonuclease (DNase) (50 μg/ml; Roche) + Liberase TL (25 μg/ml; Sigma-Aldrich)] at 37°C for 25 min, with mixing at 200 rpm. EDTA was added at a final concentration of 5 mM to halt digestion. Single-cell suspensions were passed through a 70-μm filter and washed with R5 buffer [RPMI 1640 containing 5% fetal bovine serum (FBS) and 10 mM Hepes]. Red blood cells were lysed with ammonium-chloride-potassium (ACK) lysis buffer (Lonza) for 3 min at room temperature, washed, and resuspended in R5 buffer until they were stained for flow cytometry.

Single-cell suspensions were incubated in Fc Block (TruStain FcX anti-mouse CD16/32, BioLegend) for 10 to 15 min on ice and washed with PBS. Cells were stained on ice for 30 min in PBS with primary antibodies, followed by staining on ice for 30 min with a cocktail of LIVE/DEAD Fixable Blue Viability Dye (Invitrogen) and fluorescent antibodies (list of antibodies used included). Cells were washed with FACS buffer (PBS containing 2% FBS and 2 mM EDTA), followed by fixation for 15 min with Cytofix/Cytoperm solution (BD Biosciences). Cells were washed twice with Perm/Wash buffer (BD Biosciences) and stained for intracellular *Salmonella* and iNOS. A complete list of antibodies used in this study is provided in table S1. After washing, cells were resuspended in a FACS buffer and analyzed on a LSR II cytometer (Becton Dickinson). Data were acquired with DIVA software (BD Biosciences) and analyzed using FlowJo software (TreeStar).

### Ex vivo *S*Tm entry assay

Splenocyte cell suspension was prepared using Liberase TL–digested spleens from mice that have been infected with WT *S*Tm SL1344 for 1 month, similar to preparation of splenocytes described in the “Flow cytometry” section. After red blood cell lysis, splenocytes were washed twice with 10 ml of RPMI 1640 containing 10% fetal calf serum (FCS) and 10 mM Hepes (R10 buffer) and resuspended in R10 buffer at 7 × 10^6^ cells/ml. Tomato^+^ *S*Tm SL1344 was grown aerobically to stationary phase overnight at 37°C in LB supplemented with streptomycin (200 μg/ml). Bacterial broth culture was spun for 10 min at 2100*g* at room temperature (RT) to pellet bacteria, washed once in sterile PBS, and resuspended in sterile PBS. Bacteria were added to splenocytes at a multiplicity of infection of 10. One milliliter of cells and bacterial mixture were plated per well into six-well tissue culture plates and were spun at 250*g* at RT for 5 min. Plates were then placed into 37°C incubator. After 30 min, plates were spun at 350*g* at RT for 5 min. Supernatant was carefully taken off to minimize cell loss, and plates were washed once by resuspending with 1 ml of R10 buffer per well before being collected for flow cytometry to measure intracellular Tomato^+^ *S*Tm levels.

### Immunofluorescence microscopy

Spleens were harvested and frozen in optimal cutting temperature compound (Thermo Fisher Scientific), and frozen sections (8 μm in thickness) were placed on SuperFrost Plus cryosection slides (Thermo Fisher Scientific). Sections were fixed in ice-cold acetone at −20°C for 10 min and then allowed to dry. A boundary was drawn around tissue sections using a pap pen (Thermo Fisher Scientific). Sections were washed with PBS and then blocked with a staining buffer (PBS with 3% bovine serum albumin and 5% normal mouse serum) for 30 min at room temperature. After blocking, sections were stained with the primary antibodies in a staining buffer for 2 hours at room temperature. Sections were washed and then stained for 2 hours at room temperature with fluorescent-conjugated secondary antibodies. A complete list of antibodies used in this study is provided in table S1. Slides were washed in PBS and then mounted using ProLong Diamond (Life Technologies). Images were acquired on a Zeiss LSM 700 or 880 confocal microscope with the ZEN 2010 software (Zeiss) and processed using Fiji software.

### Cell preparation for 10X genomics scRNA-seq

All samples subjected to scRNA-seq were prepared and FACS-enriched using the following procedures. *S*Tm-infected spleens were harvested and digested in buffer containing HBSS + Ca^2+^ + Mg^2+^ + DNase (50 μg/ml; Roche) + Liberase TL (25 μg/ml; Sigma-Aldrich) at 37°C for 25 min, with mixing at 200 rpm. EDTA was added to 5 mM final concentration to stop digestion reaction, and cells were washed with RPMI 1640 containing 10% FCS. Following red blood cell lysis, splenocytes were washed twice with RPMI 1640 containing 10% FCS. Splenocytes were stained with an antibody mixture for surface markers [CD11b Alexa Fluor 647, CD11c phycoerythrin-Cy7, Ly6C peridinin chlorophyll protein (PerCP) Cy5.5, Ly6G fluorescein isothiocyanate, CD3 allophycocyanin (APC) efluor 780, CD19 APC efluor 780, and NK1.1 APC efluor 780] for 25 min on ice, washed twice with RPMI 1640 containing 10% FCS, and then resuspended in the same buffer with 1:2000 4′,6-diamidino-2-phenylindole. Splenocytes were then FACS-enriched on a BD FACSAria cell sorter. A permissive gating strategy was used to simultaneously enrich CD11b^+^CD11c^+^Ly6C^+^ MΦs and capture other splenocytes for sequencing. Sorting gates were set tightly for size/scatter, singlet, and living cells but more loosely for CD3/CD19/NK1.1^−^, CD11b^+^, Ly6G^−^, Ly6C^+^, and CD11c^+^ cells as shown in fig. S1B. A complete list of antibodies used in this study is provided in table S1. The viability of sorted cells was checked using Trypan blue staining and hemocytometer inspection under a light microscope. Samples had viability greater than 90%. Cells were resuspended to a concentration of 500 to 1200 cells/μl, partitioned, and captured for sequencing on a 10× Chromium Controller. Libraries were prepared by the Stanford Functional Genomics Facility (SFGF) using the 10X Genomics 3′ GEX v3.1 Kit and sequenced on the Illumina HiSeq4000 platform to a depth of ~40,000 to 50,000 reads per cell. Raw sequencing data were demultiplexed by SFGF to yield fastqs reads.

### Sequencing alignment and data preprocessing

Paired-end reads were mapped to *Mus musculus* genome reference GRCm38 using 10X Cell Ranger (version 3.1.0) with parameters “--expect-cells = 10,000 --chemistry = auto.” Spliced and unspliced read counts were estimated by velocyto (v0.17.17) in “run10x” mode. We performed downstream preprocessing and analyses on the UMI count matrices estimated by Cell Ranger. To eliminate low-quality cells, we kept cells that had more than 500 detected genes and 1000 UMI read counts. We removed any stressed cells that had greater than 5% of total UMI counts that mapped to the mitochondrial genome. In total, we identified 22,512 cells (WT + Δ*steE S*Tm-infected mice) and 9892 cells (WT *S*Tm-infected mice treated with isotype control and anti-TNF antibodies) for downstream analysis. UMI counts were normalized for sequencing coverage such that each cell has a total number of counts equal to the median library size of all cells, yielding counts per median (CPM). The normalized CPM was added with a pseudocount of 1 and log_2_-transformed. Scanpy package (v1.6.0) was used to perform data preprocessing and data transformation. RNA velocity was inferred using scVelo (v0.2.2) with default parameters in “dynamical” mode.

### Cell clustering and cell type annotation

The SAM algorithm (version 0.8.5) was run with default parameters (*36*). SAM outputs gene weights, principal components, a nearest-neighbor graph, and two-dimensional Uniform Manifold Approximation and Projections were used for visualization, on which the processed gene expression data can be also overlaid. We used the Leiden algorithm (version 0.8.4) (*64*) to determine the number of clusters in the entire immune population and inferred the immune cell type based on the approach proposed by PanglaoDB (*33*). Briefly, a cell type–specific score was calculated for each cluster using a vector of cell type–specific genes and their associated weight

$$S_{j,k}=\frac{\sum_{i=1}^{N}Z_{k,j,i}\cdot w_{i}}{\sqrt[{3}]{N}}$$ (1)

where *S*_*j*,*k*_ is the cell-type activity (CTA) score for cell type j in cell cluster k and *N* is the total number of marker genes. *Z* is the *Z* score–standardized gene expression counts. *w_i_* is the gene weight associated with gene *i* in the given cell type, which is provided by PanglaoDB marker gene set. *N* is the set size of marker genes in cell type j. For a given cell cluster, CTA scores are then ranked from highest to lowest, and the top ranking cell type is selected as the “winner.” We manually inspected the winner cell type and determined that cluster 3 (fig. 2B) was misannotated as “γδ T cells” because it did not express any T cell receptor γ, a definitive marker for γδ T cells. Because cluster 3 expresses mostly myeloid lineage genes, we annotated it as MNP 1.

### Differential gene expression analysis

Differential gene expression was computed with a negative binomial test function (see the “Data and Materials availability” section in Acknowledgment for link to archived code on Github). We compared the expression of each gene in each cluster against the rest of the populations. FDR was calculated using the Benjamini-Hochberg (BH) procedure. Genes were identified as DEGs based on FDR, log_2_ fold change in mean expression, and % detection in the query cluster.

### Myeloid cell, MNP, and monocyte/macrophage subclustering

To ensure that we did not remove any of the MNPs (GMP, CM, or MΦ) for downstream analysis, we first subcluster the myeloid populations including dendritic cells, neutrophils, MNP 1, or MNP 2. We removed a few cells that appeared to be outliers (cell barcodes: A1_CAGCGTGAGTCTAACC-1, A2_CACTGAAAGCGCCTCA-1, B2_GAGCTGCTCCGTGCGA-1, A3_ATACCGAGTTTCGACA-1, A3_CAATGACGTTGGCCTG-1, A3_GAAGGGTTCCGCACGA-1, A3_GAGTTTGTCAGCGTCG-1, A3_GTCTCACTCGGAATGG-1, B3_AACCCAAGTTGTCATG-1, B3_GATGGAGGTGCTCTCT-1, A4_CTACAGATCCACAGGC-1, A4_TAGGGTTCAAATGATG-1, B4_GTCACGGTCAGCCCAG-1) and any cell that expressed Pou6f1 > 1 log_2_ CPM, a transcription factor typically detected in the mouse brain (*65*, *66*). We removed genes that were detected >1 UMI count in less than 20 cells and then preprocessed the data using SAM preprocessing function with the parameters “norm = ftt” and “filter_genes = false.” We then executed the algorithm with the parameter “preprocessing = StandardScaler” to yield the myeloid subpopulations in Fig. 1D. Leiden clustering was executed with parameter “resolution = 0.9.” We manually annotated cell types based on the DEGs in each cluster.

### MNP subclustering

To further analyze CM and MΦ, we removed cells that were annotated as GMP in the MNP subpopulations (Leiden clusters 4, 8, 9, and 10 in Fig. 1D) and then preprocessed the data using SAM preprocessing function with the parameter “filter_genes = false.” We executed the algorithm with default parameters to yield the monocyte/MΦ subpopulations (Fig. 3A). Leiden clustering was executed with parameter “resolution = 1.5.” We manually identified cell identities based on the expression level of myeloid marker genes and DEGs in each MΦ subpopulation.

### Differential representation test on cell state

To test statistical association between infection conditions and cell state frequency, we stratified the dataset by experimental pairs (i.e., WT *S*Tm-infected mice 1 paired with Δ*steE S*Tm-infected mice 1) and computed the chi-square distribution of counts for each condition and cell state. This ensures that we are contrasting cell state frequency in each condition while taking into account experimental variation due to sample processing or other technical factors. This test is also known as the Cochran-Mantel-Haenszel (CMH) test (*67*, *68*). To illustrate the procedure of CMH test, we quantify the CMH test statistic for a cell state A (i.e., Ace^+^, 11 MΦs). We first define *n*_A,*i*_ as the number of cells that belong to cell state A, and *n*_A*'*,*i*_ as the number of cells in the complement set (not part of cell state A) in the first condition, where *i* is the index of the experimental strata across all *K* pairs of conditions (i.e., WT *S*Tm-infected mice *i* versus Δ*steE S*Tm-infected mice *i*). Similarly, we define *m*_A,*i*_ and *m*_A',*i*_ as the set size of cell state A and its complement in the second condition, respectively. The total sum of all the cells across the two conditions in the index *i* is denoted as *T_i_*. We can then quantify the CMH test statistic as

$$\chi_{\mathrm{CMH}}^{2}=\frac{{\left\{\sum_{i=1}^{K}\left[n_{A,i}-\frac{\left(n_{A,i}+n_{A^{\prime },i})(n_{A,i}+m_{A,i}\right)}{T_{i}}\right]\right\}}^{2}}{\sum_{i=1}^{K}\frac{\left(n_{A,i}+n_{A^{\prime },i})(m_{A,i}+m_{A^{\prime },i})(n_{A,i}+m_{A,i})(n_{A^{\prime },i}+m_{A^{\prime },i}\right)}{T_{i}^{2}(T_{i}-1)}}$$ (2)

Under the null hypothesis, there is no association between condition and cell state frequency for each stratum. The test statistic should then asymptotically follow a chi-square distribution. We accounted for multiple hypothesis testing by calculating the FDR with BH procedure. Cell state with greater than twofold difference in common odds ratio and less than 0.05 FDR between conditions was considered statistically significant.

### Gene set overrepresentation analysis

To identify functional pathways that were enriched more than expected by chance, we performed GSOA on a list of DEGs between cells of interest. Only DEGs that had an FDR < 0.05, log_2_ fold change > 0.25, and >25% detection were considered as input for GSOA. We measured the fraction of DEGs that belonged to each pathway under MSigDB Hallmark gene sets and computed the significance in overlap with hypergeometric test. The set size was set to the total number of mouse genes annotated in GRCm38 (31,053 genes). FDR was calculated as before using BH procedure. Gene sets with an FDR < 0.1 were considered statistically significant in enrichment.

### Ensemble expression score analysis

Score is calculated as per “score_genes” function in Scanpy with the provided gene list. Briefly, this is the average expression of the set of input genes subtracted by the average expression of a randomly sampled set of genes, controlled for expression range. A positive score indicates that the average expression for the selected genes is above the group of randomly selected reference genes across the entire population.

### Cell cycle analysis and annotation

To predict the cell cycle phase of individual single cells, we retrieved a list of cell cycle–related genes (S and G_2_-M) (*69*). We then used the “score_genes_cell_cycle” function in Scanpy package to annotate the cell cycle phase of each cell.

## Notes

View/request a protocol for this paper from *Bio-protocol*.
