# Supplementary material for: Single-cell profiling identifies ACE+ granuloma macrophages as a nonpermissive niche for intracellular bacteria during persistent Salmonella infection
Source: Sci Adv. 2023 Jan 6;9(1):eadd4333. doi: 10.1126/sciadv.add4333 (PMC9821941; doi:10.1126/sciadv.add4333)
Supplement: Supplementary file 1 — Figs. S1 to S6 Table S1 [file sciadv.add4333_sm.pdf]

Science Advances
AAAS

Supplementary Materials for

Single-cell profiling identifies ACE$^+$ granuloma macrophages as a nonpermissive niche for intracellular bacteria during persistent Salmonella infection

Trung H. M. Pham *et al.*

Corresponding author: Denise M. Monack, dmonack@stanford.edu; Trung H. M. Pham, tpham8@stanford.edu; Stephen R. Quake, steve@quake-lab.org

Sci. Adv. **9**, eadd4333 (2023)
DOI: 10.1126/sciadv.add4333

This PDF file includes:

Figs. S1 to S6
Table S1

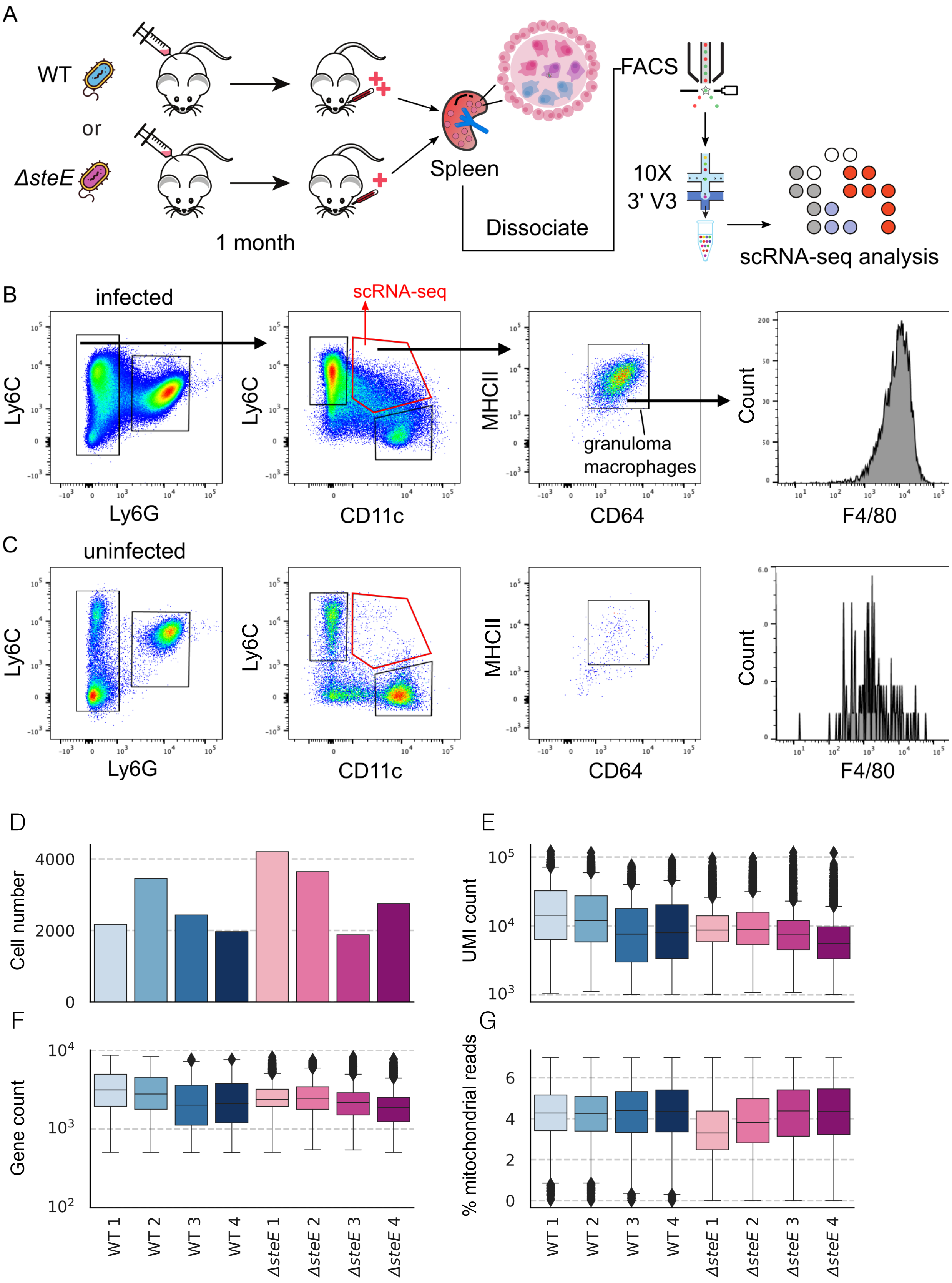
A
WT
or
ΔsteE
1 month
Spleen
Dissociate
FACS
10X
3' V3
scRNA-seq analysis
B
infected
scRNA-seq
Ly6C
Ly6G
CD11c
MHCII
CD64
granuloma
macrophages
Count
F4/80
C
uninfected
D
Cell number
E
UMI count
F
Gene count
G
% mitochondrial reads
WT 1
WT 2
WT 3
WT 4
ΔsteE 1
ΔsteE 2
ΔsteE 3
ΔsteE 4

Fig. S1.

Related to Figure 1 (A) schematic outline of the droplet-based scRNA-seq of splenocytes from mice infected either with WT *S*Tm or *ΔsteE* *S*Tm. **(B-C)** FACS gating scheme for $CD11b^{+}CD11c^{+}Ly6C^{+}CD64^{+}MHC^{hi}$ granuloma macrophages, which also have high F4/80 expression. Samples were gated for size/scatter, singlet, living $CD11b^{+}$ population then further gated as displayed. Plots shown are results from WT-*S*Tm infected. For permissive FACS enrichment of $CD11b^{+}CD11c^{+}Ly6C^{+}$ granuloma macrophages, sorting gates were set tightly for size/scatter, singlet, and living cells but more loosely for CD11b, Ly6G, Ly6C, and CD11c markers. This enrichment strategy enables significant enrichment of granuloma-associated macrophages while simultaneously capturing the full spectrum of immune cells in the infected spleens. Red arrow indicates the $CD11b^{+}CD11c^{+}Ly6C^{+}$ mononuclear phagocyte (MNP) population targeted for enrichment for scRNA-seq library preparation. **(D)** Number of analyzed cells that passed the quality controls from individual mice. Cells that had < 1000 unique molecular identifier (UMI) counts, < 500 detected genes, and > 5% reads with mitochondrial origin (indicate stressed or dying cells) were filtered. A total of 22,512 cells from WT and *ΔsteE*-*S*Tm infected mice passed quality controls. **(E)** Distribution of UMI counts in analyzed cells. **(F)** Distribution of the number of detected genes in analyzed cells. **(G)** Distribution of the percent reads that are mapped to mitochondrial genes in analyzed cells.

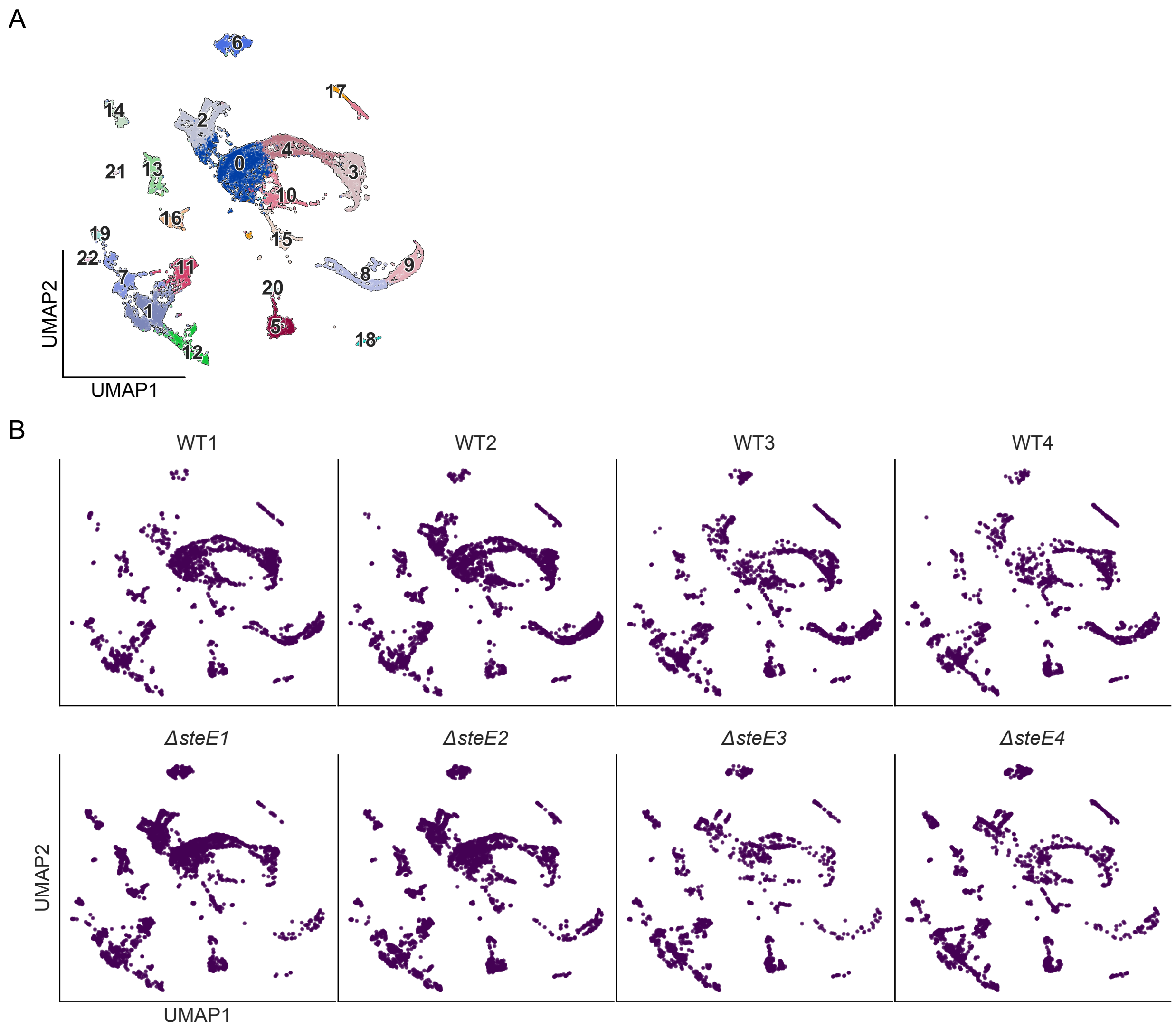

Fig. S2.

Related to Figure 1. (A) UMAP projection colored by Leiden clusters. **(B)** UMAP projections of cells from either WT *S*Tm or *ΔsteE S*Tm-infected mice. Cells are well mixed in each part of the manifold, indicating a lack of technical batch effect.

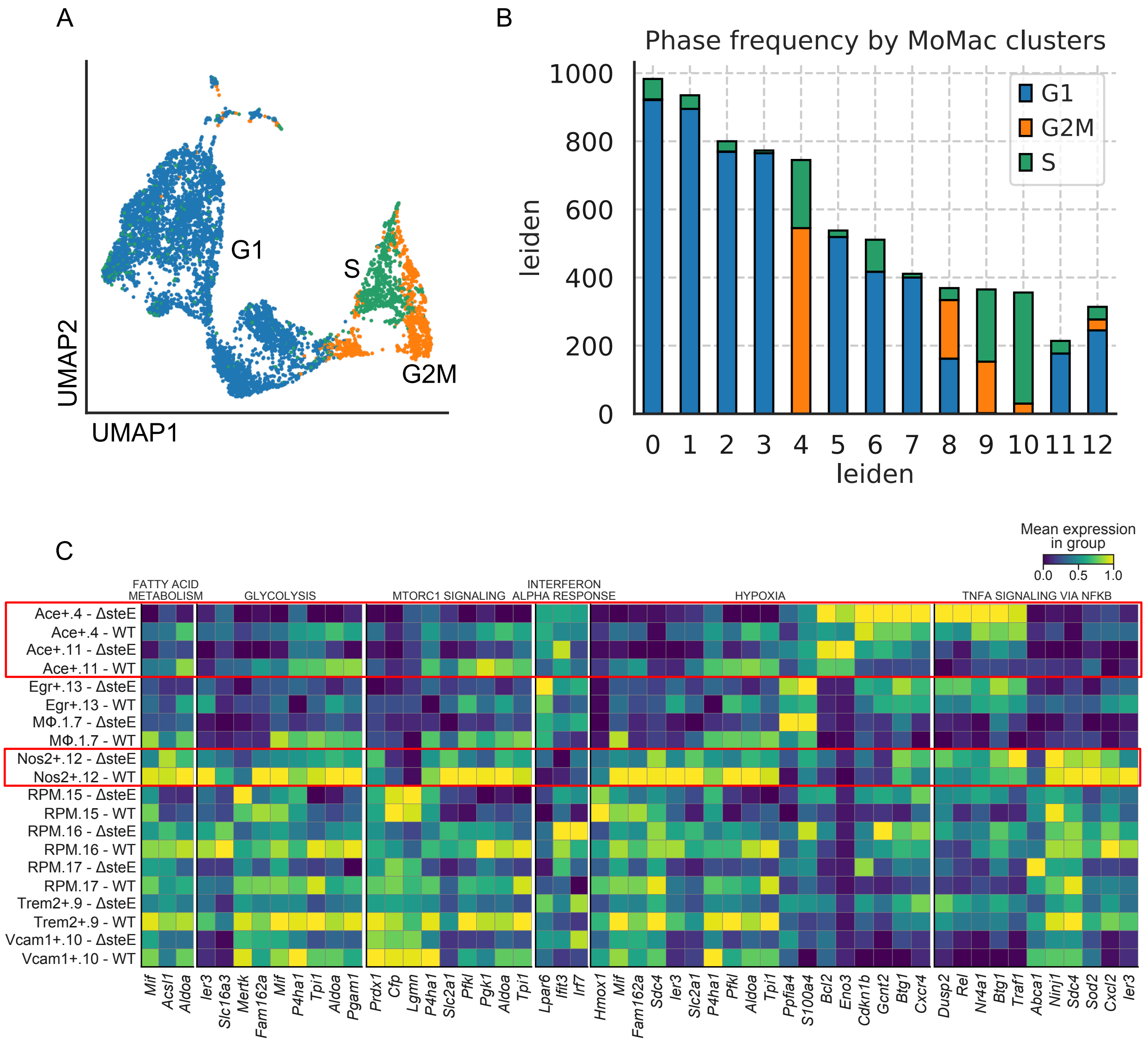

Fig. S3.

Related to Figure 3. (A) UMAP projection of myeloid populations colored by predicted cell cycle states. **(B)** Cell cycle phase frequency for each MNP cluster. **(C)** Heatmap of standardized expression of genes in selected pathways in Figure **3H**.

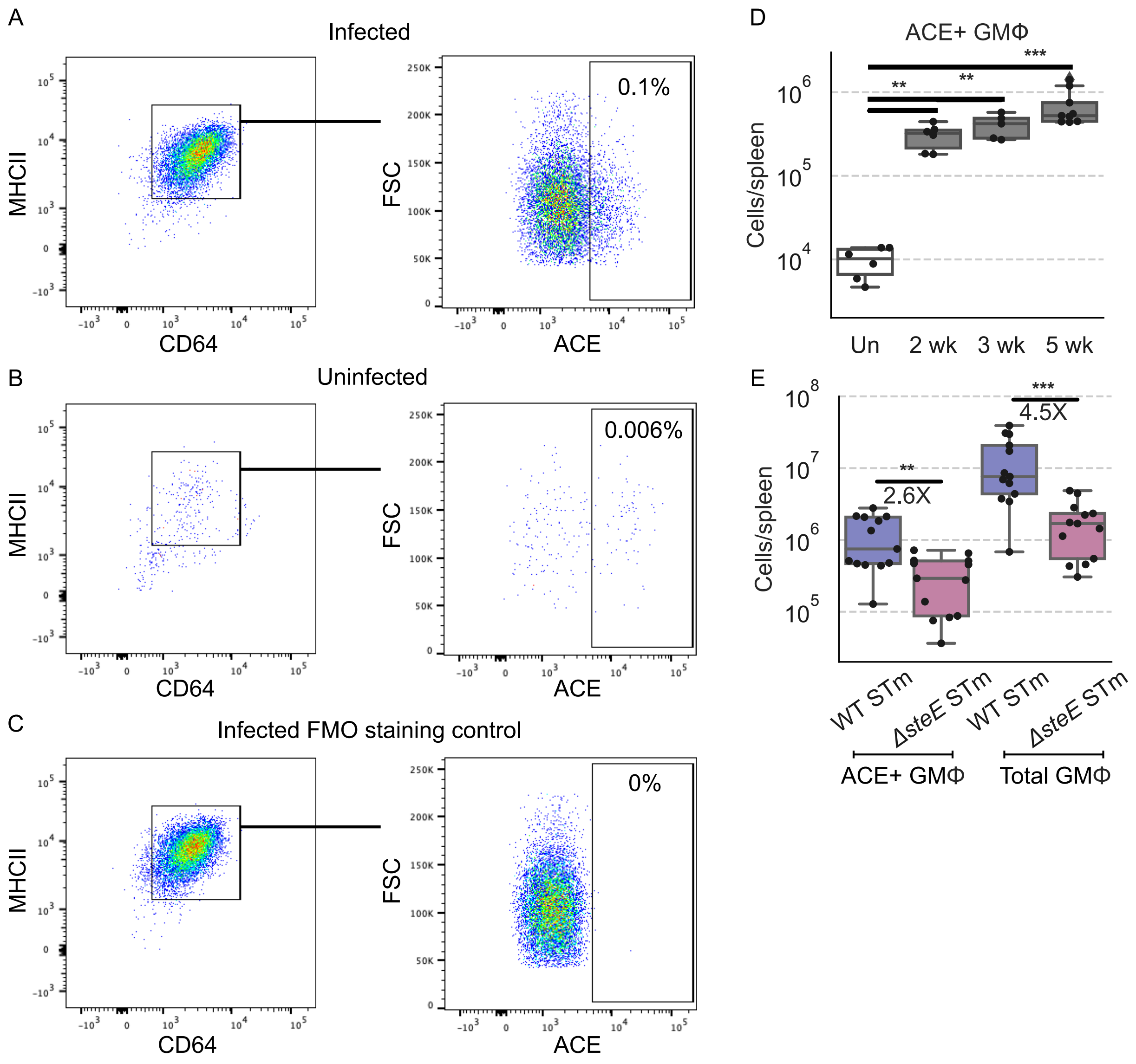

Fig. S4.

Related to Figure 4. Mice were infected intraperitoneally with 2 x 10^3 CFU WT-*S*Tm and analyzed at 1 month post-inoculation. Flow cytometry gating for ACE$^+$ granuloma macrophages. Splenocytes were gated for singlet, living CD11b$^+$CD11c$^+$Ly6C$^+$ population, then gated as displayed. Percentages indicate frequencies of gated cells among total splenocytes. Plots shown are results from infected mice **(A)**, uninfected mice **(B)**, or infected mice but without ACE antibody stain as a control **(C)**. **(D)** The number of ACE$^+$ granuloma macrophages (GMΦ) isolated from the spleens of uninfected mice or mice that had been infected with WT *S*Tm for 2, 3, or 5 weeks. At least 6 mice were included in each group. Dots: individual mice. **(E)** The number of

ACE$^+$ GMΦ or total GMΦ isolated from the spleen of mice that had been inoculated with WT or *ΔsteE* STm for 1-month. Thirteen mice were included in each group. Dots: individual mice. Numbers below the significance bar indicate the fold-change between WT *S*Tm and *ΔsteE* *S*Tm infection groups. **(D-E)** Significance calculated using a two-tailed Mann-Whitney test. ** $p < 0.01$, *** $p < 0.001$.

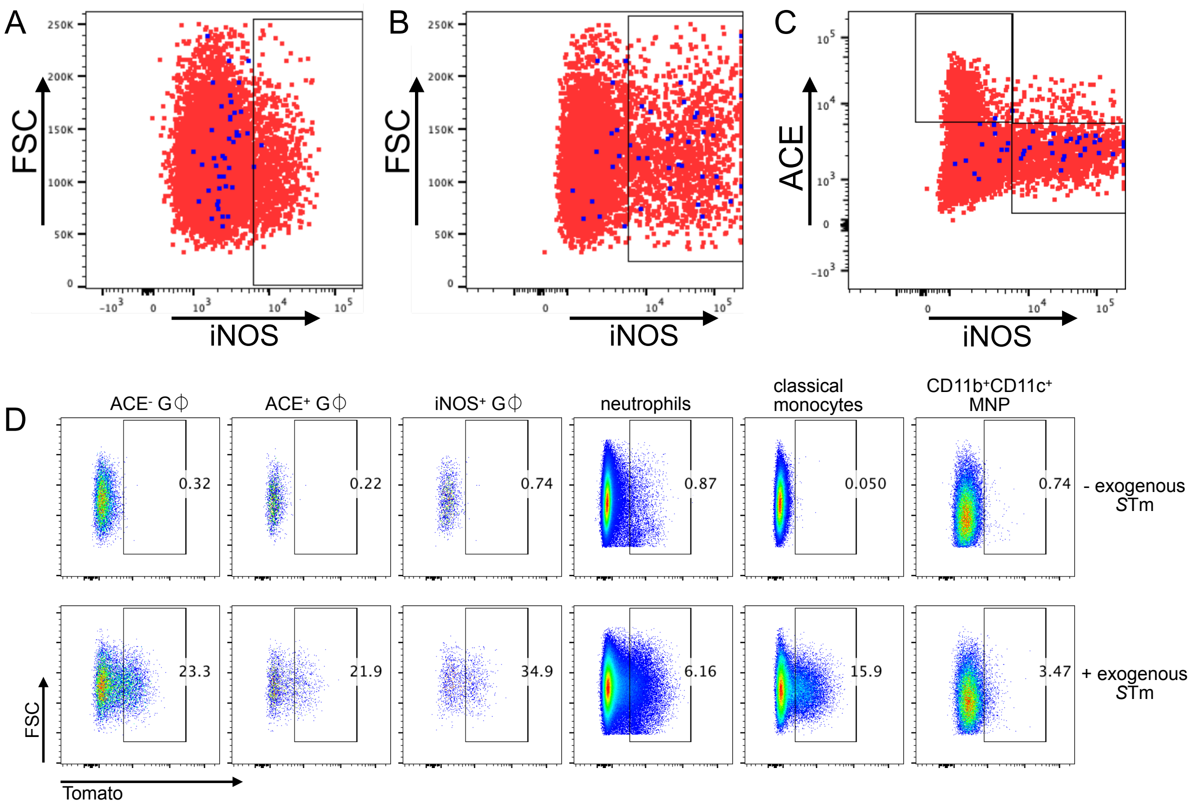

Fig. S5.

Related to Figure 5. (A-C) Mice were infected *intraperitoneally* (*i.p.*) with 2 x 10^3 CFU WT-*S*Tm and analyzed at 1 month post-inoculation. Flow cytometry showing *S*Tm-containing cells among $iNOS^+$ and ACE^+ granuloma macrophages. $CD11b^+CD11c^+Ly6C^+CD64^+MHC^{hi}$ granuloma macrophages were gated as in Figure S1B, then plotted for FSC, iNOS, or ACE as shown. Red events: all granuloma macrophages, blue events: granuloma macrophages harboring intracellular *S*Tm. **(D)** *Ex vivo* measurements of *S*Tm entry into splenocytes from mice that had been infected for 1-month. Gated frequencies indicate entry of $Tomato^+$ *S*Tm added exogenously *ex vivo* into different cell populations by FACS analysis. Abbreviations: granuloma macrophage, GΦ; $CD11b^+CD11c^+$ mononuclear phagocyte, $CD11b^+$ $CD11c^+$ MNP.

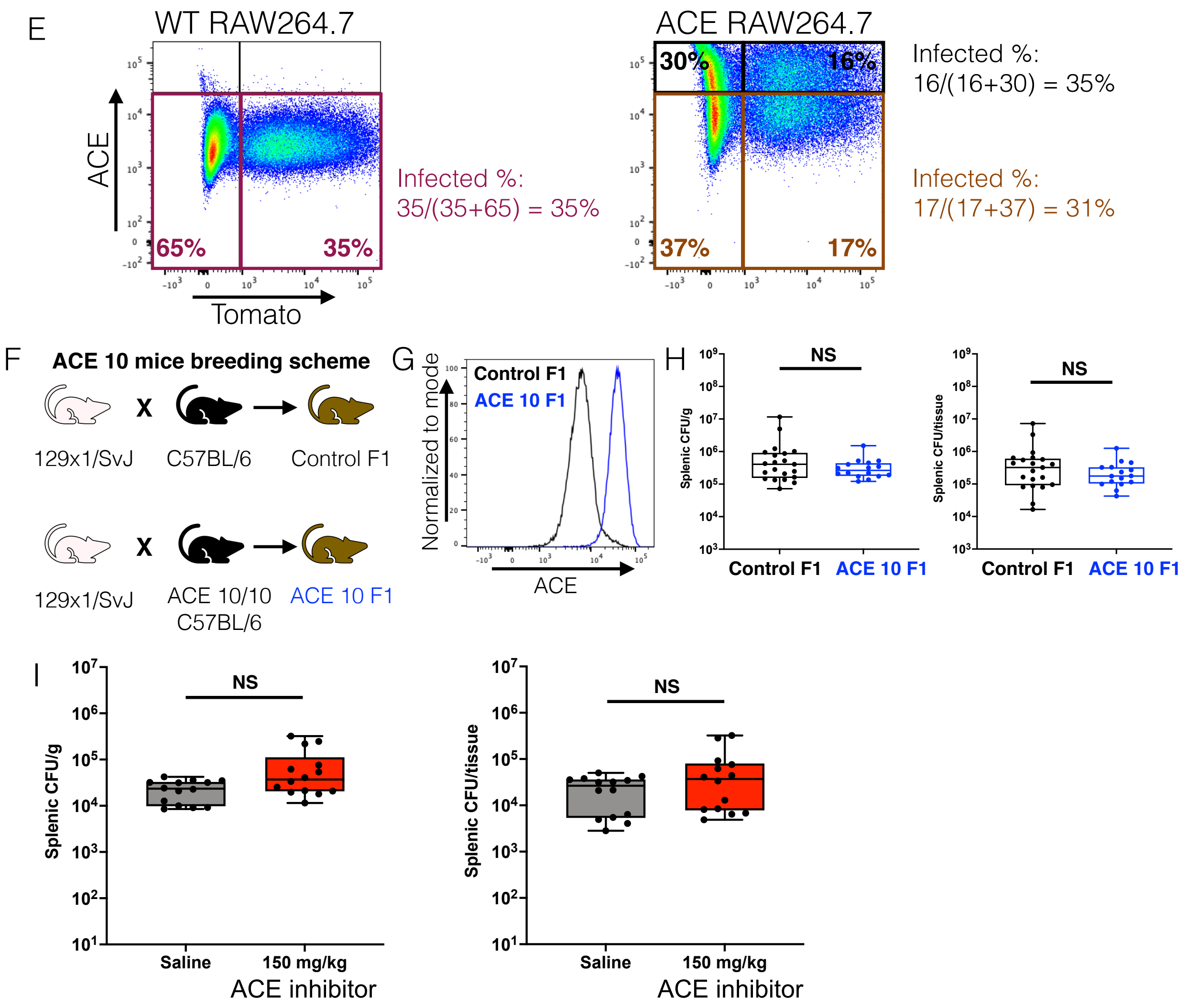

Fig. S5 continued.

Related to Figure 5. (E) *Ace* was expressed in RAW264.7 macrophages using lentiviral transduction. WT and ACE-overexpressing RAW264.7 macrophages were infected with *S*Tm and analyzed by flow cytometry 20 hours later. The percent frequencies of $S\text{Tm}^+$ cells were similar between ACE^- and ACE^+ cells. **(F)** Breeding scheme to generate [ACE 10/10 x 129x1/SvJ] F1 mice and [C57BL/6 x 129x1/SvJ] F1 control mice for infection. **(G-H)** Mice were infected with 1-2 x 10^3 CFU WT-*S*Tm *i.p.* and analyzed at two weeks post-inoculation. **(G)** Flow cytometry analysis shows splenic $\text{CD11b}^+\text{CD11c}^+\text{Ly6C}^+\text{CD64}^+\text{MHC}^{hi}$ granuloma macrophages from infected ACE 10 heterozygous F1 mice expressing significantly higher ACE levels compared to

granuloma macrophages from control F1 mice. **(H)** CFU measurements show similar *S*Tm bacterial levels in the spleens of ACE 10 heterozygous F1 mice and control mice. **(I)** Inhibition of ACE enzymatic activity *in vivo* had no significant impact on *S*Tm levels in infected spleens. Mice were infected for 1 month then treated with either saline control or 150 mg/kg of the ACE inhibitor captopril *i.p.* daily for 7 days before analysis. **(H-I)**. Dots: individual mice. Significance calculated using a two-tailed Mann-Whitney test. NS $p > 0.05$.

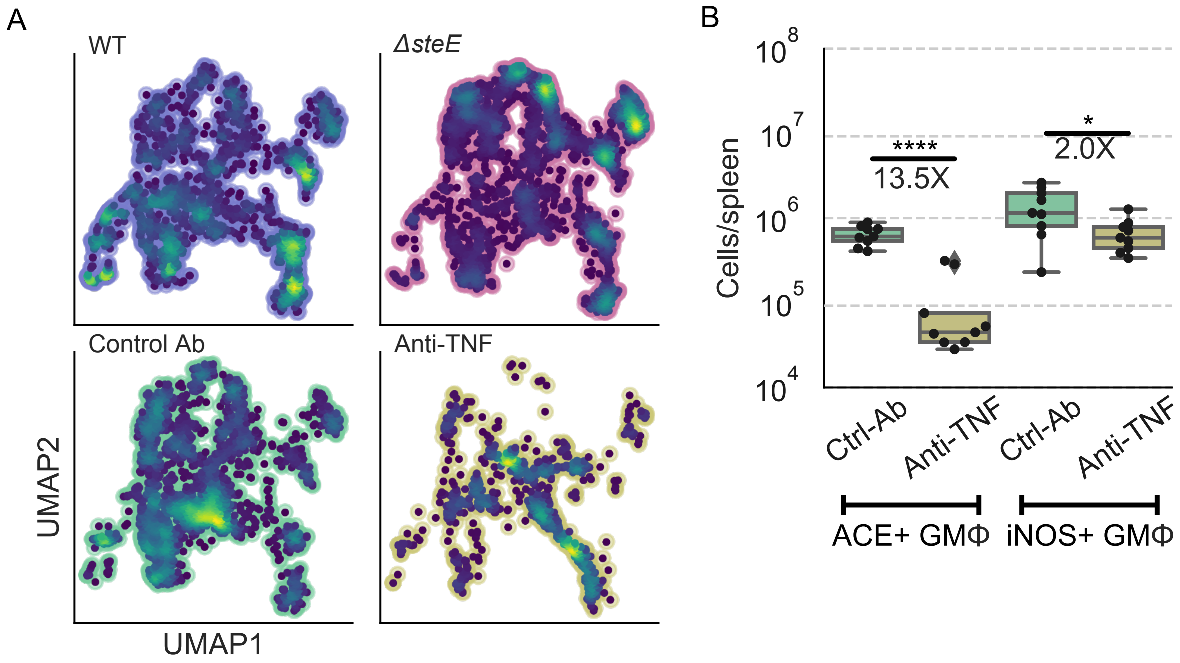

Fig. S6.

Related to Figure 6. (A) UMAP projections of mononuclear phagocytes, excluding dendritic cells, isolated from WT *S*Tm, *ΔsteE S*Tm-infected mice, or WT *S*Tm-infected mice treated with isotype control or anti-TNF. Color reflects cell density in each part of the manifold. **(B)** The number of ACE^{+} and $iNOS^{+}$ GMΦ cells isolated from the spleen of mice that had been inoculated with WT *S*Tm for 1-month and treated with isotype control (Ctrl-Ab) or anti-TNF antibody (Anti-TNF). Nine mice were included in each group. Numbers below the significance bar indicate the fold-change between Ctrl-Ab treated and Anti-TNF treated groups. **(B)**. Dots: individual mice. Significance calculated using a two-tailed Mann-Whitney test. * $p < 0.05$, **** $p < 0.0001$.

Table S1: List of antibodies used in this study

Antibody	SOURCE	IDENTIFIER
Anti-*Salmonella* CSA-1	KPL	Cat# 02-91-99
CD106 (VCAM-1) biotin	Biolegend	Cat# 105704<br>RRID:AB_313205
CD106 (VCAM-1) biotin	Biolegend	Cat# 105704<br>RRID:AB_313205
CD106 (VCAM-1) PE-Cy7	Biolegend	Cat# 105720<br>RRID:AB_2214046
CD11b Alexa Fluor 647	Biolegend	Cat# 101218<br>RRID:AB_389327
CD11b Brilliant Violet 785	Biolegend	Cat# 101243<br>RRID:AB_2561373
CD11c Alexa Fluor 700	eBioscience	Cat# 56-0114-80<br>RRID:AB_493993
CD11c FITC	eBioscience	Cat# 11-0114-82<br>RRID:AB_464940
CD11c PE-Cy7	BD Pharmingen	Cat# 558079<br>RRID:AB_647251
CD115 Brilliant Violet 605™	Biolegend	Cat# 135517<br>RRID:AB_2562760
CD143/ACE unconjugated goat antibody	R&D Systems	Cat# AF1513<br>RRID:AB_354832
CD19 APC eFluor 780	eBioscience	Cat# 47-0193-82<br>RRID:AB_10853189

CD19 BUV 395	BD Biosciences	Cat# 563557<br>RRID:AB_2722495
CD3e APC eFluor 780	eBioscience	Cat# 47-0031-82<br>RRID:AB_11149861
CD301 Alexa Fluor 647	Bio-Rad	Cat# MCA2392A647<br>RRID:AB_872012
CD301 PE	Biolegend	Cat# 145704<br>RRID:AB_2561961
CD45.1 APC	Biolegend	Cat# 110713<br>RRID:AB_313502
CD45.2 PerCP Cy5.5	eBioscience	Cat# 45-0454-80<br>RRID:AB_953592
CD49b APC eFluor 780	eBioscience	Cat# 47-5971-82<br>RRID:AB_11218895
CD64 PE	Biolegend	Cat# 139304<br>RRID:AB_10612740
CD64 Brilliant Violet 605™	Biolegend	Cat# 139323<br>RRID:AB_2629778
CD86 Brilliant Violet 510™	Biolegend	Cat# 105040<br>RRID:AB_2315766
Donkey anti-goat IgG Alexa Fluor 488	ThermoFisher	Cat# A11055<br>RRID:AB_2534102
Donkey anti-goat IgG Alexa Fluor 647	ThermoFisher	Cat# A21447<br>RRID:AB_2535864
F4/80 PE	eBioscience	Cat# 12-4801-82<br>RRID:AB_465923

F4/80 Brilliant Violet 650™	Biolegend	Cat# 123149 RRID:AB_2564589
IL-4Ra PE-Cy7	Biolegend	Cat# 144806 RRID:AB_2565599
IL-4Ra BV421	BD Biosciences	Cat# 564086 RRID:AB_2738584
iNOS Alexa Fluor 488	eBioscience	Cat# 53-5920-82 RRID:AB_2574423
iNOS APC	eBioscience	Cat# 17-5920-80 RRID:AB_2573243
iNOS e450	eBioscience	Cat# 48-5920-82 RRID:AB_2802293
Ly-6C APC	eBioscience	Cat# 17-5932-82 RRID:AB_1724153
Ly-6C PerCP/Cy5.5	eBioscience	Cat# 45-5932-82 RRID:AB_2723343
Ly-6G FITC	BD Biosciences	Cat# 551460 RRID:AB_394207
Ly-6G BUV395	BD Biosciences	Cat# 563978 RRID:AB_2716852
MerTK APC eFluor 780	eBioscience	Cat# 47-5751-82 RRID:AB_2848373
MHC class II APC eFluor 780	eBioscience	Cat# 47-5321-80 RRID:AB_1548792
MHC class II Alexa Fluor 700	eBioscience	Cat# 56-5321-82 RRID:AB_494009

Ultra-LEAF™ Purified Rat IgG1, κ Isotype	Biolegend	Cat# 400458
Ultra-LEAF™ Purified anti-mouse TNF	Biolegend	Cat# 506348<br>RRID:AB_2616672
